# Positioning of an unprecedented spiro[5.5]undeca ring system into kinase inhibitor space

**DOI:** 10.1038/s41598-020-78158-9

**Published:** 2020-12-04

**Authors:** Arramshetti Venkanna, Lalita Subedi, Mahesh K. Teli, Prema Dhorma Lama, Bhargav Gupta Nangunuri, Sang-Yoon Lee, Sun Yeou Kim, Mi-hyun Kim

**Affiliations:** 1 Gachon Institute of Pharmaceutical Science and Department of Pharmacy, College of Pharmacy, Gachon University, Yeonsu-gu, Incheon, Republic of Korea; 2 Gachon Advanced Institute for Health Science and Technology, Graduate School and Neuroscience Research Institute, Gachon University, Yeonsu-gu, Incheon, Republic of Korea

**Keywords:** Drug discovery, Chemistry, High-throughput screening, Virtual drug screening

## Abstract

In-house 1,5-oxaza spiroquinone **1**, with spiro[5.5]undeca ring system, was announced as an unprecedented anti-inflammatory scaffold through chemistry-oriented synthesis (ChOS), a chemocentric approach. Herein, we studied how to best position the spiro[5.5]undeca ring system in kinase inhibitor space. Notably, late-stage modification of the scaffold **1** into compounds **2a-r** enhanced kinase-likeness of the scaffold **1**. The improvement could be depicted with (1) selectivity with target shift (from JNK-1 into GSK-3) and (2) potency (> 20-fold). In addition, ATP independent IC_50_ of compound **2j** suggested a unique binding mode of this scaffold between ATP site and substrate site, which was explained by docking based optimal site selection and molecular dynamic simulations of the optimal binding site. Despite the shift of kinase profiling, the anti-inflammatory activity of compounds **2a-r** could be retained in hyperactivated microglial cells.

## Introduction

### Chemistry-oriented synthesis of 1,5-oxaza spiroquinone with a spiro[5.5]undeca ring system

**Figure 1:**
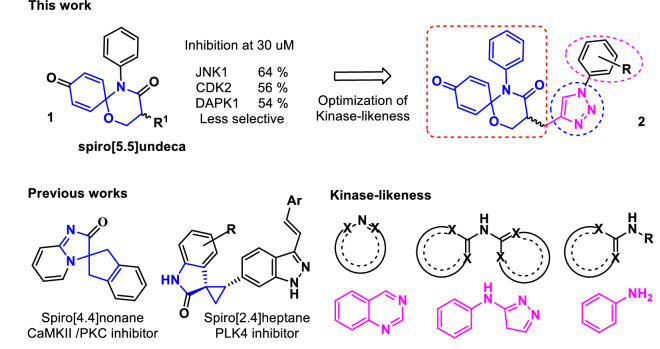
The positioning of 1,5-oxaza spiroquinone **1** in kinase inhibitor space.

It is challenging for researchers to increase the occupation of artificial drug space in the enormity of chemical space^1–4^. How many structurally diverse molecules are synthesizable for both selective and effective targeting in drug discovery? Because current approaches (of informatics and medicinal chemistry) commonly use the target as their query, this question cannot be the top priority for drug discovery or cannot be considered without coupling from SAR. Therefore, it is inefficient for a researcher to focus on structural novelty without selecting a target molecule or a target disease. However, neither rational drug design nor virtual screening can consider (or predict) structural novelty beyond the existing dataset. Notably, unprecedented scaffolds, frameworks or ring systems will remain unidentified treasures for drug discovery^3,4^. Our research group has developed organic synthetic methodologies for increasing synthesizability of rare drug scaffolds^5–7^ and has synthesized unprecedented drug scaffolds using the developed methodologies without an initial target^9,10^. We named the strategy chemistry-oriented synthesis (ChOS)^9,10^. An unprecedented drug scaffold, 1,5-oxaza spiroquinone **1**, was also acquired through the ChOS approach^10^. The target deconvolution of scaffold **1** in kinase space showed weak inhibitory activity but suggested its potential as a selective inhibitor (Fig. 1)^10^. In addition, the spiro[5.5]undeca ring system of the scaffold **1**, which showed in vitro and in vivo efficacy, was first in kinase inhibitor space. Therefore, the results encouraged us to further investigate better positions of the scaffold in kinase inhibitor space.

### Explored molecules in kinase inhibitor space

**Figure 2:**
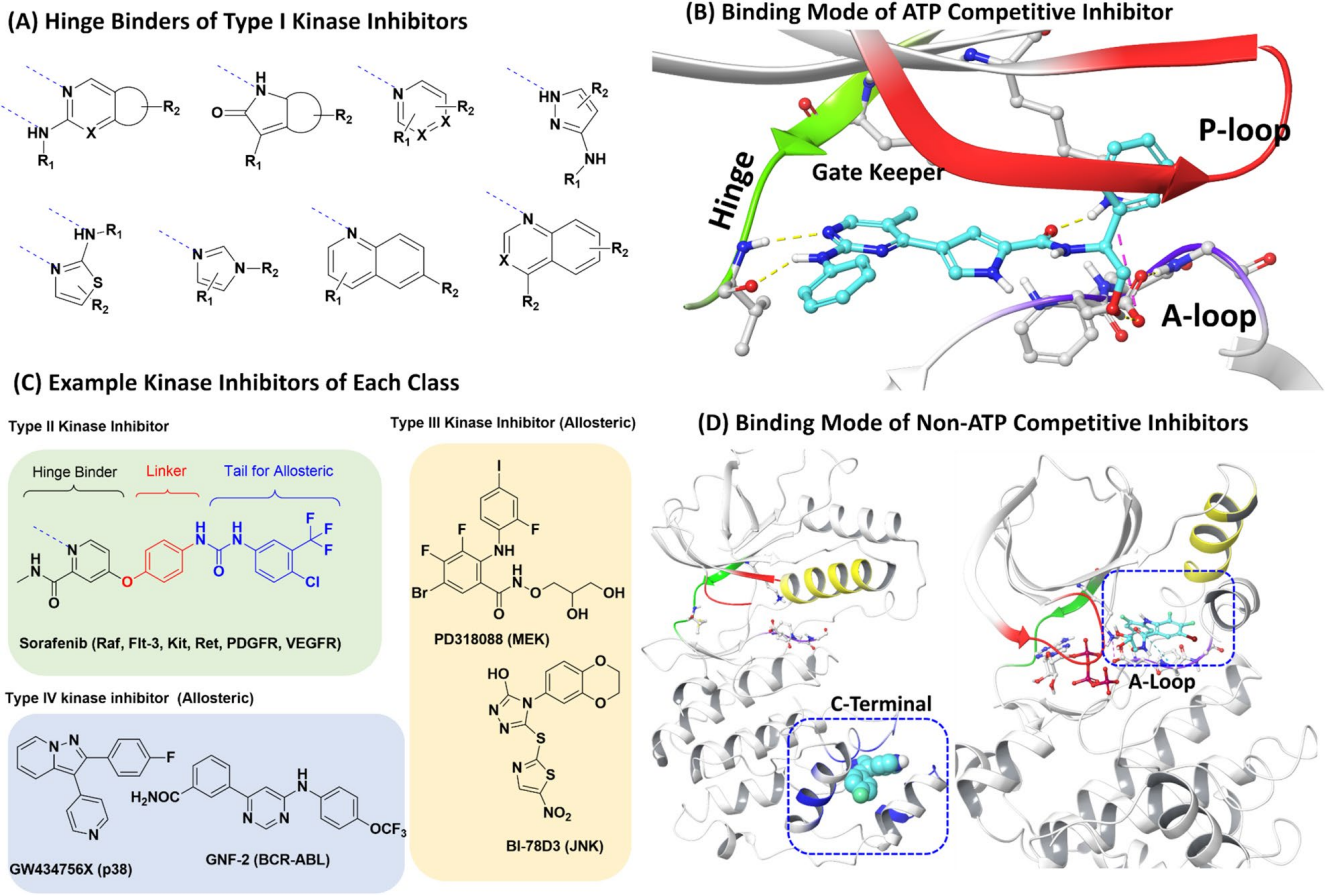
Classification of kinase inhibitors^11–27^. (**A**) Typical ATP competitive inhibitors, (**B**) the binding mode of ATP competitive inhibitors, (**C**) example kinase inhibitors of each class, and (**D**) the binding mode of non-ATP competitive inhibitors. The left of (**D**) is p38 complex with GW434756X (type IV inhibitor in the C-terminal domain). The right of (**D**) shows the MEK complex with PD318088 (a type III inhibitor between the p-loop and activation loop). The gate keeper (green), hinge (green), DFG residues of the A-loop (violet), P-loop (red), helix (yellow), and C-terminal domain (blue) are shown.

High throughput screenings for protein kinases have found notable hit compounds similar to the ATP adenine group, so that most approved kinase inhibitors possess heteroaromatic rings such as quinazoline, indolinone, pyrrolopyrimidine, pyrazolopyrimidine, or quinolone group (Fig. 2A,B)^11–14^. Furthermore, the inhibitors occupy ATP binding sites, such as binding to the adenosine group^15^, and achieve high selectivity and nanomolar affinity through structure-based rational design, which was used to control the the delicate interactions with the hinge, the gate keeper, and the sites near the p-loop and the activation loop (especially the DFG residue of the activation loop)^15–17^. Following these orthosteric inhibitors (type I), type II kinase inhibitors have also been developed through additional DFG motif occupancy (as an allosteric site) as well as the binding sites of type I inhibitors^16^. The structures of type II kinase inhibitors typically consist of a hinge binder (for type I kinase), a linker (with H-bonds), and a hydrophobic tail that can interact with the DFG motif (Fig. 2C)^18^. Thus, type II inhibitors also generally have heteroaromatic rings. In parallel with their discovery, non-ATP competitive inhibitors have also been developed through the investigation of their binding sites (Fig. 2C,D)^19,20^. While a part of the inhibitors are peptides or peptidomimetics similar to kinase substrates (or scaffold proteins)^21^, some kinases have diverse small molecule inhibitors, revealing competition with substrates (type III inhibitor) or scaffold proteins (a type IV inhibitor, remote from the ATP site)^22^. For example, among MAP kinases, JNK and p38 possess non-ATP competitive inhibitors, BI-78D3 (JIP binding site)^23^, and GW434756X (C-terminal)^24^, respectively (see. Left of Fig. 2D for GW434756X). PD318088 (right panel of Fig. 2D), trametinib and cobimetinib are non-ATP competitive inhibitors for MEK ^25^. In addition, GNF2 for BCR-ABL is a type IV inhibitor ^26^. A type V bivalent bisubstrate kinase inhibitor has been developed through a long tether between the two domains^27^. Because allosteric sites exist in less conserved parts of kinases, these non-ATP competitive inhibitors present more structural diversity than type I or II inhibitors in terms of shape, ring systems, and the relative placement of the heteroatoms (Fig. 2C). When considering such a variety of kinase inhibitors, glycogen synthase kinase 3 (GSK-3) inhibitors are notable examples showing such structural diversity because GSK-3 has seven binding sites that have been kinetically characterized^28^. The multiple binding sites are relevant to how GSK-3 can have diverse binding mechanisms with over 100 known endogenous substrates^29^. Several non-ATP competitive inhibitors with diverse ring systems have been reported^30–39^. However, despite the diversity, a GSK-3 inhibitor with a spiro ring system had not been reported until our study. In the whole kinase inhibitor space, spiro ring systems are of limited use for constructing adenine mimetic rings such as oxyindoles: spiro[2. 4]hepta (e.g., PLK-4 inhibitor)^40^ and spiro[4. 4]nonane ring systems (e.g., ZSET1446 as a CaMKII /PKC inhibitor)^41^ as shown in Fig. 1.

Obviously, the spiro[5.5]undeca core can introduce an additional plane into the two planes of 1,5-oxaza spiroquinone **1**. The angles between the three planes (rings) are (1) 79.6° between the quinone and oxaza ring, (2) 66.5° between the quinone and N-phenyl ring, and (3) 49.3° between the oxaza ring and N-phenyl ring. Such a spatial arrangement with a rigid conformation is unlike to the known inhibitors shown in Fig. 2 and is rarely observed in kinase inhibitors, although some inhibitors derived from natural products show such spatial arrangements through congested quaternary carbon centers. In the case of the quinone group, it is not a privileged scaffold of kinase inhibitors, but approximately 100 tests against 22 kinases include a 1,4-quinone substructure. Specifically, the quinone group is useful for designing covalent inhibitors such as pyranonaphthoquinone natural products showing irreversible AKT1 inhibition (type 3 inhibitor)^42^.

In this study, ring systems that have been neither synthesized nor tested as kinase inhibitors are referred to as ‘NE ring system (not yet existing ring systems)’. we introduce a spiro[5.5]undeca ring system as an NE ring system, describe how to modify the initial scaffold **1** into compounds **2** to achieve the best positioning, and elucidate the shifted kinase selectivity with its unique binding mode between the ATP and substrate binding sites. In addition, the in vitro potencies of compounds **2** are described by a hyperactivated microglia model.

## Results

### Late-stage modification of 1,5-oxaza spiroquinone 1

**Scheme 1:**
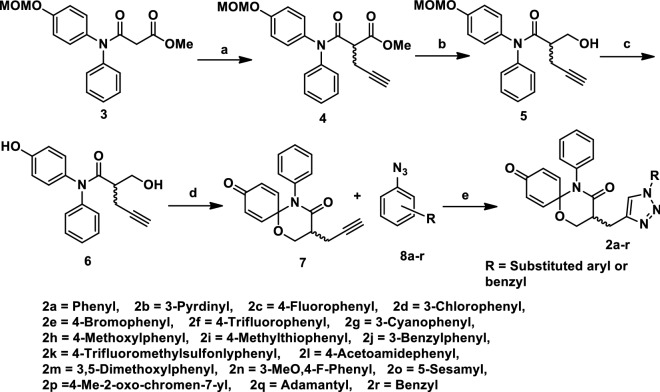
Reagents and conditions: (**a**) NaH, propargyl bromide, dry DMF, 0 °C-rt, 4 h, 92%; (**b**) LiAl(O^t^Bu)_3_H, dry THF, − 15 °C-rt, 4 h, 82%; (**c**) BF_3_^.^Et_2_O, DMS, 0–15 °C, 10–20 min, 88%; (**d**) PhI(CF_3_CO_2_)_2_, anhydrous K_2_CO_3_, NO_2_Me, 0 °C–25 °C, 4 h, 60%; (e) CuSO_4_, Sodium Ascorbate, 0 °C- rt, THF:H_2_O, 12 h.

In fragment based drug design, nonpolar atoms are typically used instead of polar heteroatoms to grow fragments^43^. Such a strategy cannot disrupt the potential binding of a pharmacophore to a target, so this fragment growing strategy was benchmarked to modify 1,5-oxaza spiroquinone **1**. For this purpose, we should also consider late-stage functionalization for efficient introduction of diverse substituents in the very last steps of the synthesis^44,45^. As shown in Fig. 1 and Scheme 1, the α-position of tertiary amide, which is the starting point for fragment growth, is the most suitable for late stage diversification compared to other substitution positions. Privileged Bemis Murcko frameworks of kinase inhibitors suggested the introduction of arylamine or heteroaromatic groups into the R1 position^17^. Therefore, compounds **2**, obtained by merging oxaza spiroquinone **1** with *N*-benzyl- or *N*-aryl-1,2,3-triazole derivatives, could be designed based on the kinase-likeness concept. Compound **7** was synthesized from malonamide ester **3** and used conditions identical to those we reported previously except for a modification of the oxidative cyclization^10^. The general conditions of the hypervalent iodine (III)-mediated oxidative cyclization (PIFA, anhydrous K2CO3, dry ACN, 0–25 °C) rely on the synthetic skill and maintaining rigorously anhydrous conditions, or else the isolated yields can decrease less than 10%. Undesirable side reactions include (1) oxidative removal of the quinone groups and (2) hydride or hydroxyl group transfer into the quinone iminium intermediate instead of the beta hydroxyl group. 1,1,1,3,3,3-Hexafluoro-2-propanol, a preferred solvent for hypervalent iodine chemistry, did not influence the inefficiency of the reaction. A base (e.g., K_2_CO_3_) was essential to prevent acid catalytic decomposition of the intermediate in general conditions. According to our recent study on the solvent effect of C-H functionalization of trivalent iodine^7,8^, nitromethane presented consistently the product of 60% isolated yield without any base. After improving the cost-effectiveness and synthetic efficiency of the reaction, aromatic azides **8a-r** were synthesized according to the literature protocol^46^. The Huisgen 1,3 dipolar cycloaddition reaction of the two click partners, **7** and **8a-r** regioselectively produced desirable products **2a**-**r** in good yields with CuSO4 as the catalyst and in the presence of sodium ascorbate (see Supplementary S1)^47^. All of the synthesized compounds were purified by column chromatography, and the structure of all synthesized compounds was characterized using MS, IR, ^1^H NMR, and ^13^C NMR techniques.

### Kinase specificity and inhibitory effect of 1,5-oxaza spiroquinone 2

**Figure 3:**
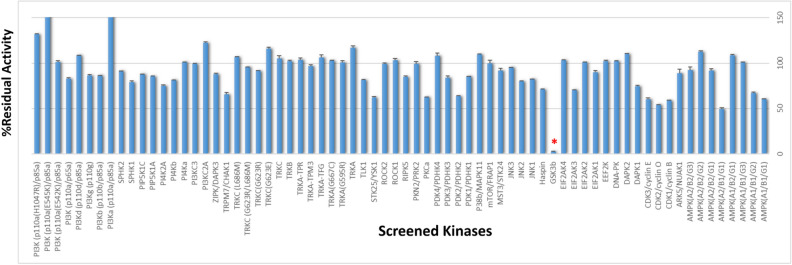
Kinase panel profiling of compound **2d**. The assay conditions of 70 kinase panels are described in supplementary S7. Red asterisk indicates selective full inhibition of GSK-3β. Average activity is shown as histogram chart (blue) with standard deviation bar (black).

After synthesizing compounds **2a-r**, compound **2d** was randomly chosen for the comparison with compound **1c** in kinome space. To navigate kinome space, among all typical protein kinases (No. of kinases: 369), compound **2d** was first tested for kinases that were inhibited by compound **1c**. Specifically, the classes of human protein kinases that were inhibited by compound **1c** led to the selection of thirty-three kinases from eight classes (TK, CMGC, CAMK, AGC, CK1, STE, TKL, and others). For example, CDK family and JNKs were chosen from CMGC. PKC, PKN and ROCK were selected from AGC, and DAPKs and NUAK1 also were selected from CAMK. Secondly, atypical kinases screening was conducted, and this step included seventeen lipid kinases and twenty non-eukaryotic kinases, as shown in Fig. 3 and Supplementary S7. Surprisingly, among a total of 70 kinases, compound **2d** perfectly inhibited only GSK-3β (more than 90% activity), as shown in Fig. 3 and Table1. Obviously, while compound **1c** did not show any inhibitory activity on GSK-3β, compound **2d** fully inhibited GSK-3β. In the case of CMGC, AGC, and CAMK kinase, the two compounds showed slightly different inhibition for DAPK1 and JNK1, and CDK family members were similarly inhibited by the two compounds. Based on these promising results, the inhibitory activities of eighteen late-stage synthetic compounds (**2a-r**) were tested on GSK-3β, as shown in Table2. The steric effects of the substituents, the electronic effects of the substituents, and the substitution positions were considered in the investigation. First, compounds with an *N*-benzyl group (compound **2r**) and an *N*-phenyl group (compound **2a**) showed similar activities. Thus, flexible benzyl derivatives were not considered for further study. Second, the ortho, meta, and para positions of the *N*-benzene group did not show consistent results. For example, a meta-benzyl group (compound **2j**) was superior to compound **2a,** but the para-methoxy (compound **2h**) and para-thiomethoxy (compound **2i**) groups did not cause enhanced activity compared to that of compound **2a**. Small substituents (compound **2c**) or electron-withdrawing groups (compound **2f**) at the para position also did not offer any improvement over compound **2a**. Meta-substituted compounds **2d** (meta-Cl) and **2g** (meta-CN) showed better activity than compound **2a**, which encouraged us to further study the meta position. Heteroaromatic rings did not offer any improvement over compound **2b**.

**Table 1:** Comparison of chosen kinase panel profiling between compound **1c** and compound **2d**.

Kinase	% Residual Activity at 30 μM^a^	
	Compound 2d	Compound 1c^b^
ARK5/NUAK1	88.9 ± 6.7	70.8 ± 0.1
CDK1/cyclin B	59.0 ± 0.5	46.0 ± 0.8
CDK2/cyclin O	54.3 ± 0.1	43.1 ± 1.0
CDK3/cyclin E	60.3 ± 1.9	59.3 ± 1.2
DAPK1	74.6 ± 1.7	45.4 ± 0.1
DAPK2	110.2 ± 0.6	120.1 ± 0.6
GSK3β	**2.2 ± 0.8**	**107.5 ± 1.9**
Haspin	70.9 ± 0.9	66.4 ± 0.5
JNK1	82.7 ± 0.1	35.8 ± 0.3
JNK2	80.0 ± 0.4	58.3 ± 0.7
JNK3	95.4 ± 0.1	72.0 ± 2.0
MST3/STK24	91.8 ± 3.4	58.3 ± 0.7
P38b/MAPK11	109.9 ± 0.5	87.0 ± 1.1
PKCa	62.6 ± 0.5	45.6 ± 1.2
PKN2/PRK2	99.5 ± 3.3	63.5 ± 1.8
RIPK5	85.0 ± 1.6	59.3 ± 1.0
ROCK1	103.3 ± 3.1	97 ± 2.9
ROCK2	99.4 ± 1.5	103.9 ± 2.4
STK25/YSK1	62.2 ± 1.6	66.9 ± 0.1
TLK1	81.7 ± 0.5	58.0 ± 0.9
TRKA	117.3 ± 2.6	98.3 ± 0.1
TRKB	102.2 ± 1.3	94.1 ± 1.7
TRKC	105.4 ± 3.9	97.9 ± 0.2
ZIPK/DAPK3	87.9 ± 1.3	101.8 ± 0.8

^a^Commonly tested kinases in compound **2d** and compound **1c** were displayed from the test of compound **2d** on 70 kinases.

^b^The result was also reported in our previous study^10^.

**Table 2:**
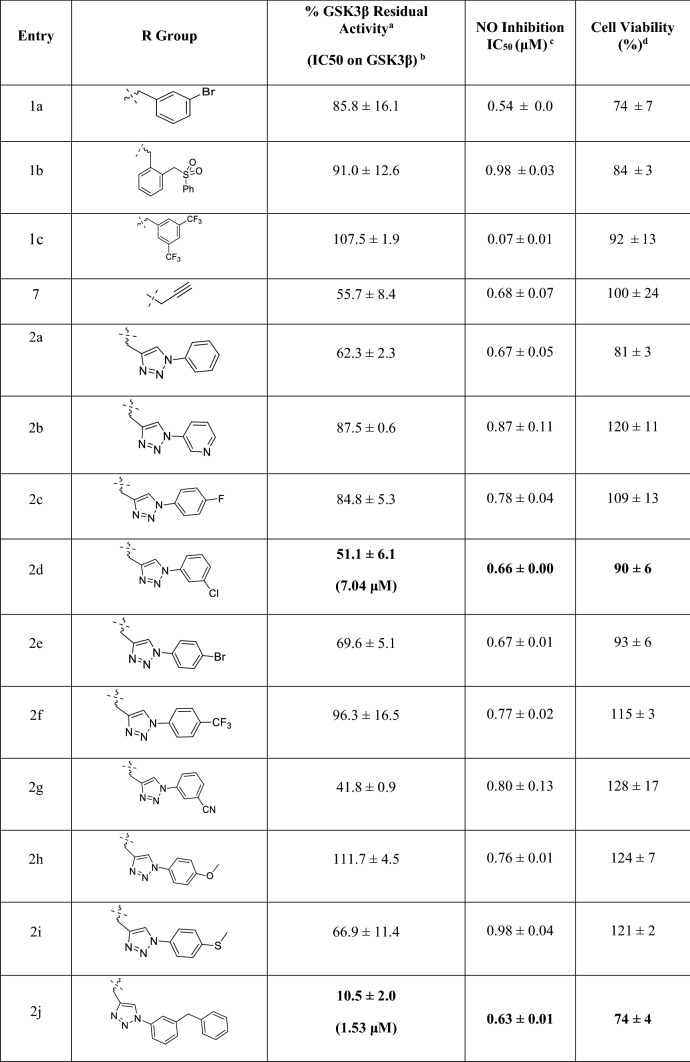

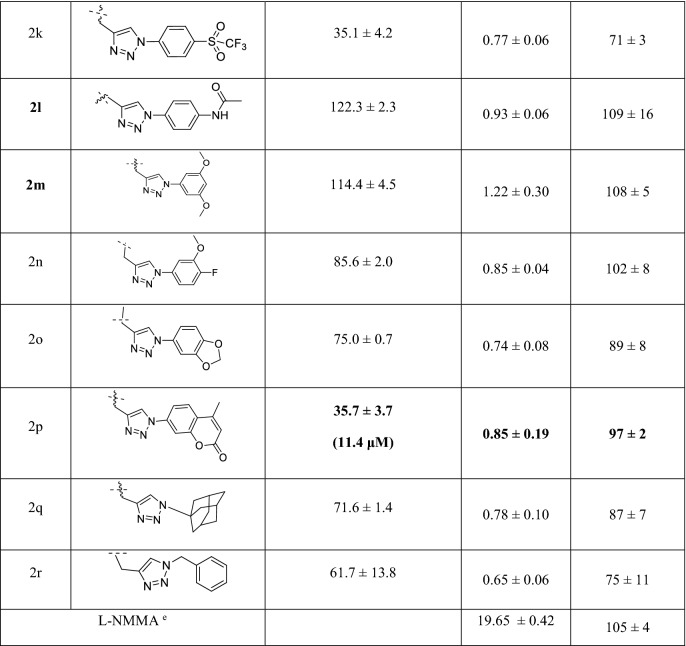
Inhibitory effect on the % GSK3β activity of 1,5-oxaza spiroquinone compounds (**1a-c**, **2a-r**) and anti-inflammatory effect on LPS-induced BV-2 cells.

^a^% GSK3β Residual Activity at 10 μM.

^b^IC50 (GSK3β) was measured at ATP (1 μM).

^c^IC_50_ value of each compound was defined as the concentration (μM) that caused 50% inhibition of NO production in LPS-activated BV-2 cells.

^d^cell viability after treatment with 5 μM of each compound was determined by MTT assay, and it is expressed as a percentage (% of LPS). The results are averages of three independent experiments, and the data are expressed as the mean ± SD.

^e^L-NMMA was positive control.

When the electrostatic effects of substituents at the same position were compared, electron withdrawing groups at the para position did not improve the activity, except for compound **2k.** Notably, a bulky adamantyl group (compound **2q**) showed activity similar to those of compounds with benzene group (compound **1a**). The comparison between compounds **2k** and **2l** showed the complexity of the noncovalent interactions involving the para position. Activity of symmetrical di-meta substituted compound **2m** suggested different environments for the two meta positions. To further elucidate the inhibitory activity at the atomic level, a molecular docking simulation of compound **2a-r** was essential. After selecting the most potent compound **2j**, IC_50_ values were measured under five ATP concentrations (1, 10, 50, 100, and 200 µM). Surprisingly, while staurosporine showed a steep decrease in IC_50_ (5 nM vs 140 nM) corresponding to increased ATP concentration, compound **2j** retained its IC_50_ activity regardless of the ATP concentration (Table 3). It means that potency of compound **2j** didn’t depend on ATP concentration.

**Table 3:** IC_50_ Measurement of the GSK3β activity of 1,5-oxaza spiroquinone compound **2j** in several ATP concentrations.

Compound	ATP concentration (µM)				
	1	10	50	100	200
2j^a^	2.65	3.53	3.79	4.04	3.45
Staurosporine^b^	5.05	6.49	19.44	32.24	140.20

^a^Compound **2j** was tested in the 10-dose IC_50_ mode with a threefold serial dilution starting at 10 μM, and the unit of IC_50_ is µM.

^b^Staurosporine was tested in the 10-dose IC_50_ mode with fourfold serial dilution starting at 20 μM, and the unit of IC_50_ is nM.

### Molecular binding mode of selected compounds 2 on GSK-3β

**Figure 4:**
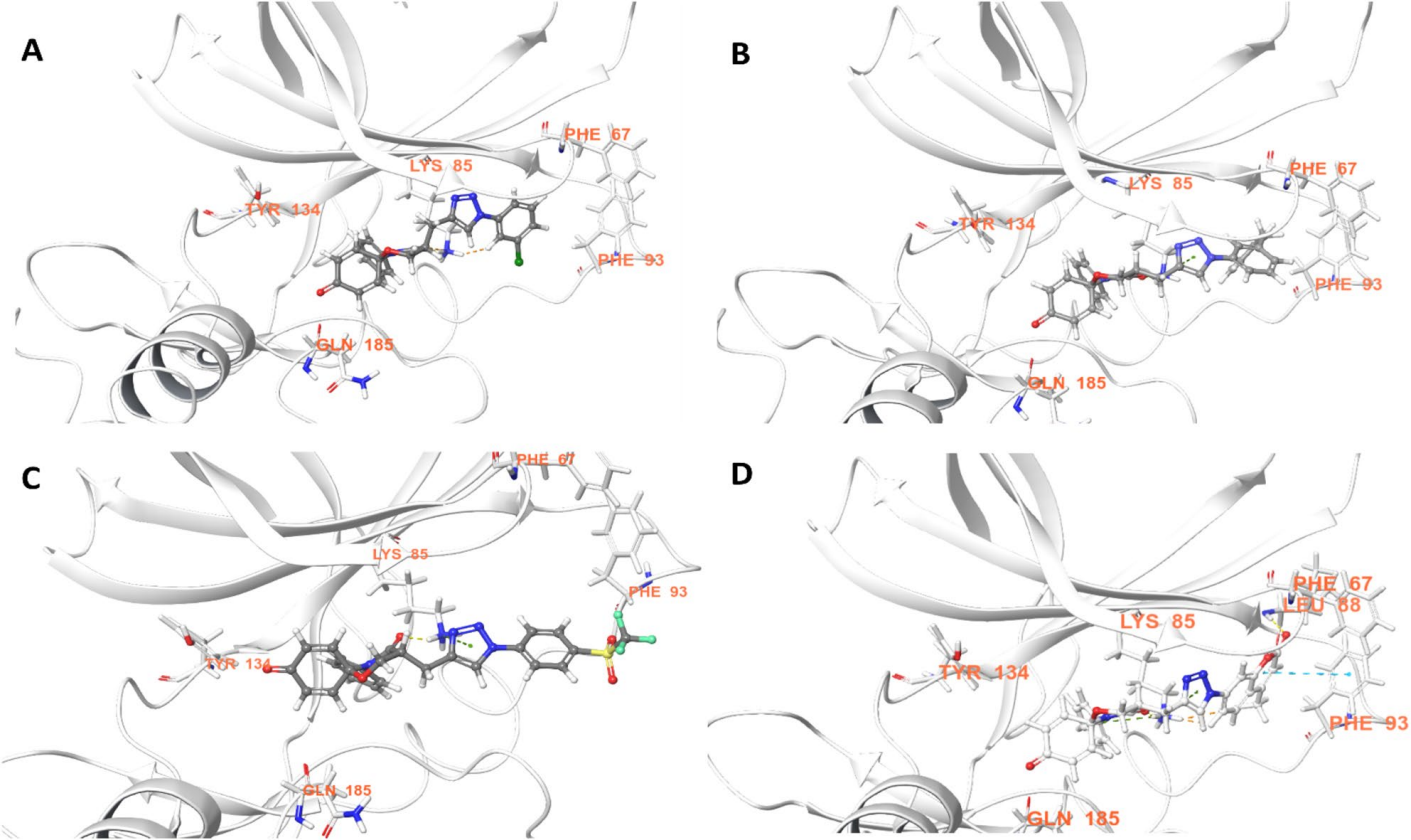
Docking simulations of selected compounds **2** at site 1 and 2 interface in 3I4B (Important interaction residues are represented with orange color text); (**A**) Interaction of compound **2d**, (**B**) Interaction of compound **2j**, (**C**) Interaction of compound **2 k**, (**D**) Interaction of compound **2p**.

Molecular docking simulations of the chosen compounds (**2d**, **2h**, **2j-m**, and **2p**) were conducted with GSK-3β crystal structures (PDB ID: 1J1B, 3I4B, 1Q3W and 1Q4L) (Tables 4–5). The logic of PDB selection was identical to Palomo et al. study^28^. These chosen compounds were used to explain the environment near to the meta-substituent (**2d** and **2m**) and the stereo-electronic effects of the para position (**2h, 2j, 2k**, and **2l**). Palomo et al. proposed six binding sites, which were reproduced for our docking models (except for axin binding site, site3)^28^. Sites 1 and 2 were partially overlapped between the ATP binding region (site 1) and the substrate binding region (site 2), so that sites1 and 2 were merged for an additional docking model to investigate binding on the interface of both binding sites. After validating the models, docking was performed to identify which of the six sites is more likely to be favored by these compounds. The binding modes of more active (**2j, 2d, 2k** and **2p**) and less active compounds (**2h, 2l** and **2m**) were iteratively compared for all probable binding sites to explore the most relevant binding site and orientation. The percentage probabilities for each binding site of each compound were calculated by counting the number of poses in each binding site among the top 1000 poses of a respective compound. The data in Table 4 suggest that the probable binding sites are site1, site 2 and site 1/2, but the data in Table 5 suggest that site1 and site 1/2 have better docking score. The data in both tables emphasize that the scaffold binds to the hinge region, which is present in two sites (site 1 and site 1/2), and suggest that the most likely binding site is the merged site, which starts from site1 and extend up to site2. In addition, site 1 cannot explain these experimental results of Table 3. In general, docking scores and experimental activity do not show complete correlation. In our case, the activity order of tested compounds except for compound **2j** roughly matched the predicted order (**2p** > **2j** > **2k** > **2m**, **2l**, and **2h**). When the docking pose of compound **2j** (or **2k**) is compared with the pose of compound **2l**, the triazole group commonly has a pi-cation interaction with Lys85 and the amide group (C=O, oxygen) of spiroquinone has additional hydrogen bonding with Lys85 in sites 1 and 2 in Fig. 4. Molecular docking calculations indicated the higher selectivity of compounds **2j, 2k, 2p,** and **2d** for the interface between sites 1 and 2 (Fig. 4, Table 4 and Table 5). The docking results of the more and less actives compounds suggested the binding preference to site 1/2 but were unable to differentiate between their bioactivity profile, which is a drawback of docking because it cannot not consider the dynamic nature of the protein.

**Table 4:** Binding site population of the Top 1000 docking poses of the selected compounds **2**.

Compound	Site 1^a^	Site 2^b^	Site 1/2^b^	Site 4^a^	Site 5^a^	Site 6^c^	Site 7^a^
2d	256	192	**320**	0	168	0	64
2h	128	192	256	0	128	0	**296**
2j	192	**360**	**384**	64	0	0	0
2k	320	**360**	**320**	0	0	0	0
2l	64	**360**	**384**	0	0	0	192
2m	**384**	232	**320**	0	0	0	64
2p	**384**	128	**320**	168	0	0	0

^a^PDB: 1J1B was used for building docking models (sites 1, 4, 5, and 7).

^b^PDB: 3I4B was used for building docking models (site 1 and sites 1 and 2).

^c^PDB: 1Q4L was used for building a docking model (site 6).

**Table 5:** Best docking score^a^ of the selected compounds **2** on each annotated binding site.

Compound	Site 1	Site 2	Site 1, 2	Site 4	Site 5	Site 6	Site 7
2d	**− 5.373**	**− **4.008	**− **4.761	0	**− **3.614	0	**− **3.119
2h	**− **4.489	**− **3.928	**− 5.438**	0	**− **3.576	0	**− **3.606
2j	**− **5.546	**− **4.019	**− 6.41**	**− **3.133	0	0	0
2k	**− **4.731	**− **3.371	**− 6.144**	0	0	0	0
2l	**− 5.617**	**− **4.925	**− **5.393	0	0	0	**− 5.798**
2m	**− **4.245	**− **4.06	**− 5.423**	0	0	0	**− **3.659
2p	**− **5.712	**− **3.165	**− 7.362**	**− **3.488	0	0	0

^a^Glide XP was selected as a scoring function with epic penalty.

**Figure 5:**
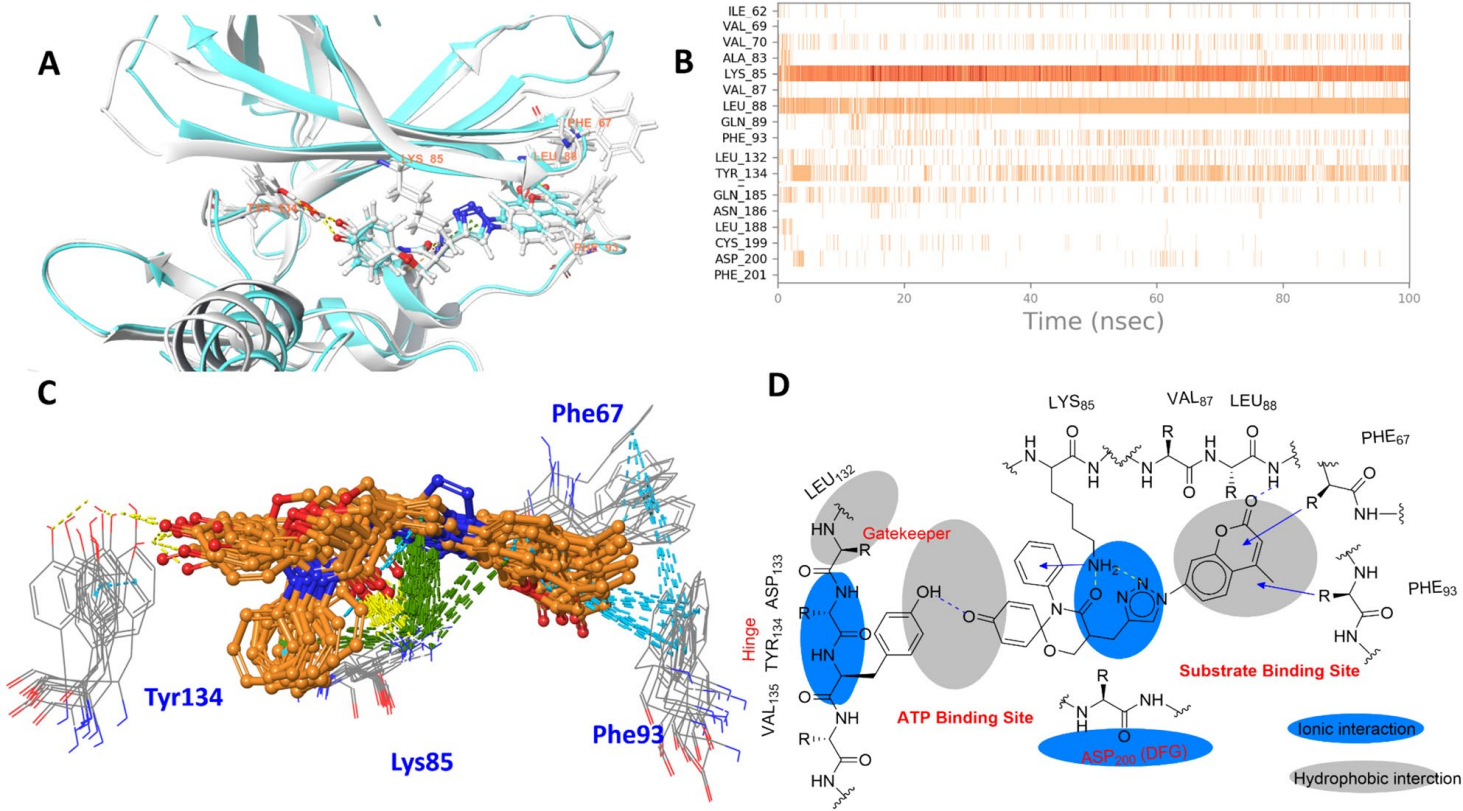
Molecular dynamics of compound **2p** at the site 1/2 interface of GSK-3: (**A**) 3D representation of Tyr134 interaction by water-bridge/H-bond, triazole anchoring by Lys85, H-bond (Leu88), and hydrophobic interaction (Phe67,93), (**B**) time dependent interaction contribution, (**C**) overlapped frames with the interactions of displayed residues showing H-bond (yellow), pi-cation (green), and pi/pi interactions (cyan), and (**D**) 2D representation of the interactions with GSK-3 showing H-bond (yellow) and pi-pi/pi-cation (arrow).

The drawback of docking was complemented by molecular dynamics (MD) simulations, and it allowed us to further explore the interaction patterns in solvated dynamic conditions at the interface of sites 1 and 2, as suggested by docking (supplementary S3 to S5). The MD simulations of the chosen docking complex (site 1 and site 1/2) provided a sample of all possible interactions in dynamic stages and validated the above binding mode indicated by molecular docking (Fig. 5). In addition, our MD results were compared with the crystallographic interaction information of ATP/substrate and their competitive inhibitors reported in the PDB. In the case of ATP competitive inhibitors (site 1), a substantial amount of X-ray data was available for comparison. Notably, site 2 is reported to bind with primed substrates at the catalytic core consisting of Phe 67 (Gly 63 to Val 69 loop) and Arg96 (Gln 89 to Arg96 loop) at the interface of the primed phosphate binding pocket (red region of Fig. 5A)^48, 49^. In contrast to the substrates, the simulations suggested that our 1,5-oxazaspiroquinone scaffold interacts partially with the catalytic core (compound **2p** of Fig. 5A)^48,50^. By comparing these results to those in the literature, we determined that the occupied regions of the chosen compounds could partially overlap with the site 1 (ATP binding site) and site 2 (substrate binding site) regions. It was repetitively observed that the triazole intermittently switched between interactions with Lys85 and Asp200 in the active site. Lys85 formed a key interaction with the ligand and anchored it to the active site for it to achieve its full potential through dynamic interactions such as π-cation, H-bonding and even hydrophobic interactions (Fig. 5B). The substitution on the N-aryl group resulted in the formation of hydrophobic interactions in the nucleotide binding region, which is surrounded by Val70, Ala83, Val110, Leu132, Tyr134, Leu188 and Cys199. Phe67 formed π–π and hydrophobic interactions with compound **2p** and played a critical role in stabilizing the chromenone substituent at the triazole (Fig. 5A,C). Even though most ATP competitive inhibitors interact with the backbone of the amino acids in the hinge region, compound **2p** formed π–π stacking and water-bridge/H-bonding interactions with the phenolic OH group of Tyr134 (Fig. 5B–D). Notably, compound **2p** presented two impressive binding characteristics: (1) the anchoring of Lys85 in the centered interaction and (2) an atypical interaction with the hinge of GSK-3 at site 1, as mentioned above. Lys85 formed the strongest interactions and was able to maintain those interactions throughout the MD simulation (Fig. 5B–C). Based on this observation, it was expected that Lys85 plays a key role in the stabilization of the binding complex through π-cation interactions with the triazole rings of compound **2** and the aromatic rings present on both sides of the triazole (Fig. 5C–D), to facilitate other noncovalent interactions at site 1 (H-bonding interactions with carbonyl groups of compounds **2**) and site 2 (hydrophobic interactions with Phe67, Val88, and Phe93).

### Anti-inflammatory effects of compounds 2

To investigate the pharmacological potential of compounds **2a-r**, they and compound **7**, a click partner, were administered to lipopolysaccharide (LPS) treated BV-2 cells as an inflammation model of hyperactivated microglia. First, nitric oxide (NO) production and cell viability were measured according to our previous study of compound **1**. The observed IC50 values of NO production are given in Table 2. Every tested compound showed a IC50 values (from 0.63 to 1.22 µM) superior to (2S)-*N*-methylarginine (L-NMMA), a well-known NOS inhibitor. The scaffold extension through click chemistry did not influence on NO production, and the IC50 values of the compounds **2a-r** were similar to those of compound **1a-b**. In the case of cell viability, the cell viabilities of some compounds slightly decreased at 5 µM but the decrease were recovered at 1 µM to solve the cell toxicity issue (Supplementary S8).

**Figure 6:**
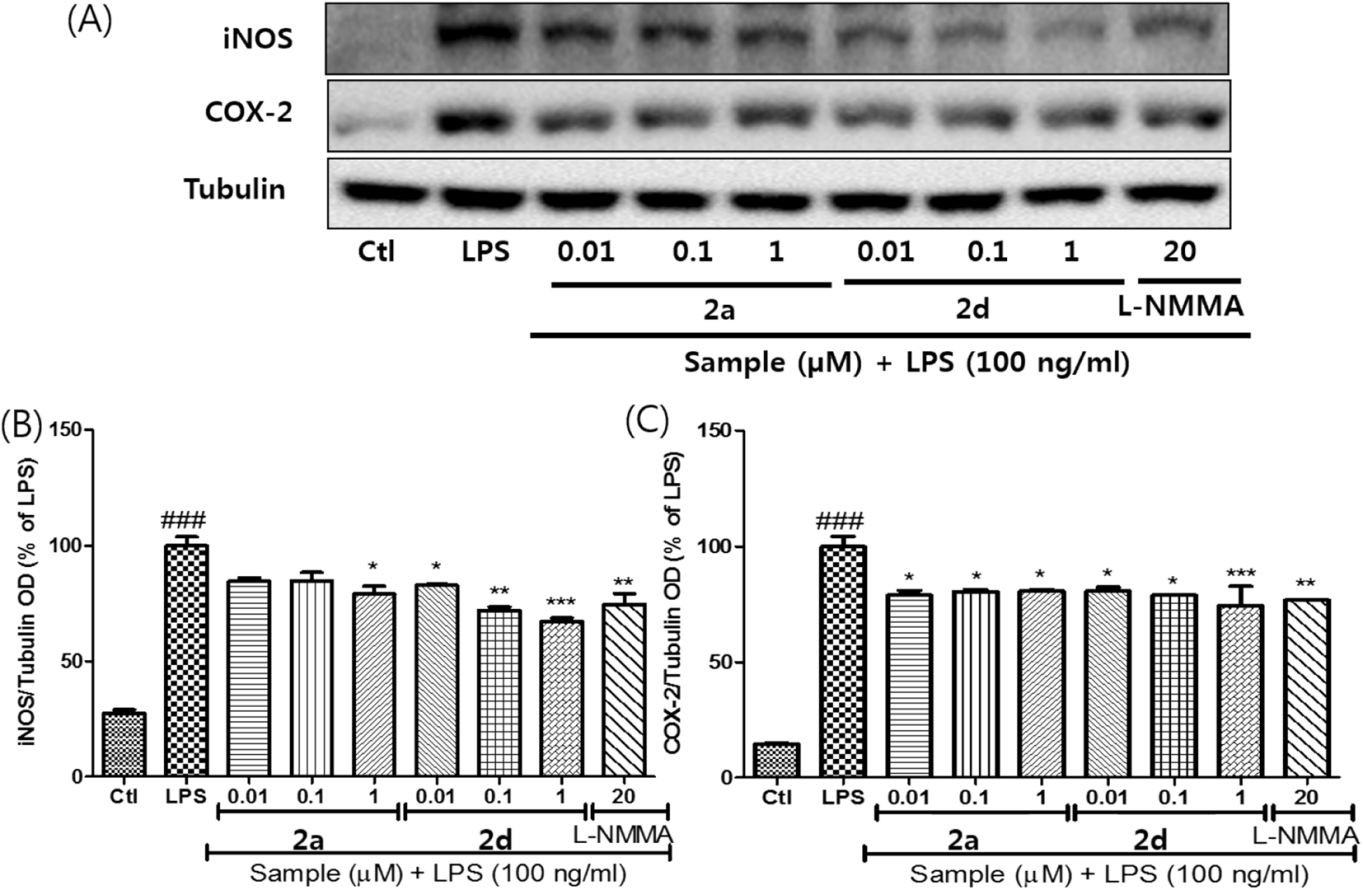
The controlled expression levels of iNOS and COX-2 by compounds **2a** and **2d** in LPS-induced BV-2 cells: (**A**) immunoblot of iNOS and COX-2, (**B**) densitometric analysis of iNOS expression, and (**C**) densitometric analysis of COX-2 expression. α-Tubulin was used as the loading control. The figures show the representative results of three independent experiments. ^###^*P* < 0.001 compared to the untreated control, *P < 0.05 and ***P < 0.001 compared to the LPS-treated group.

**Figure 7:**
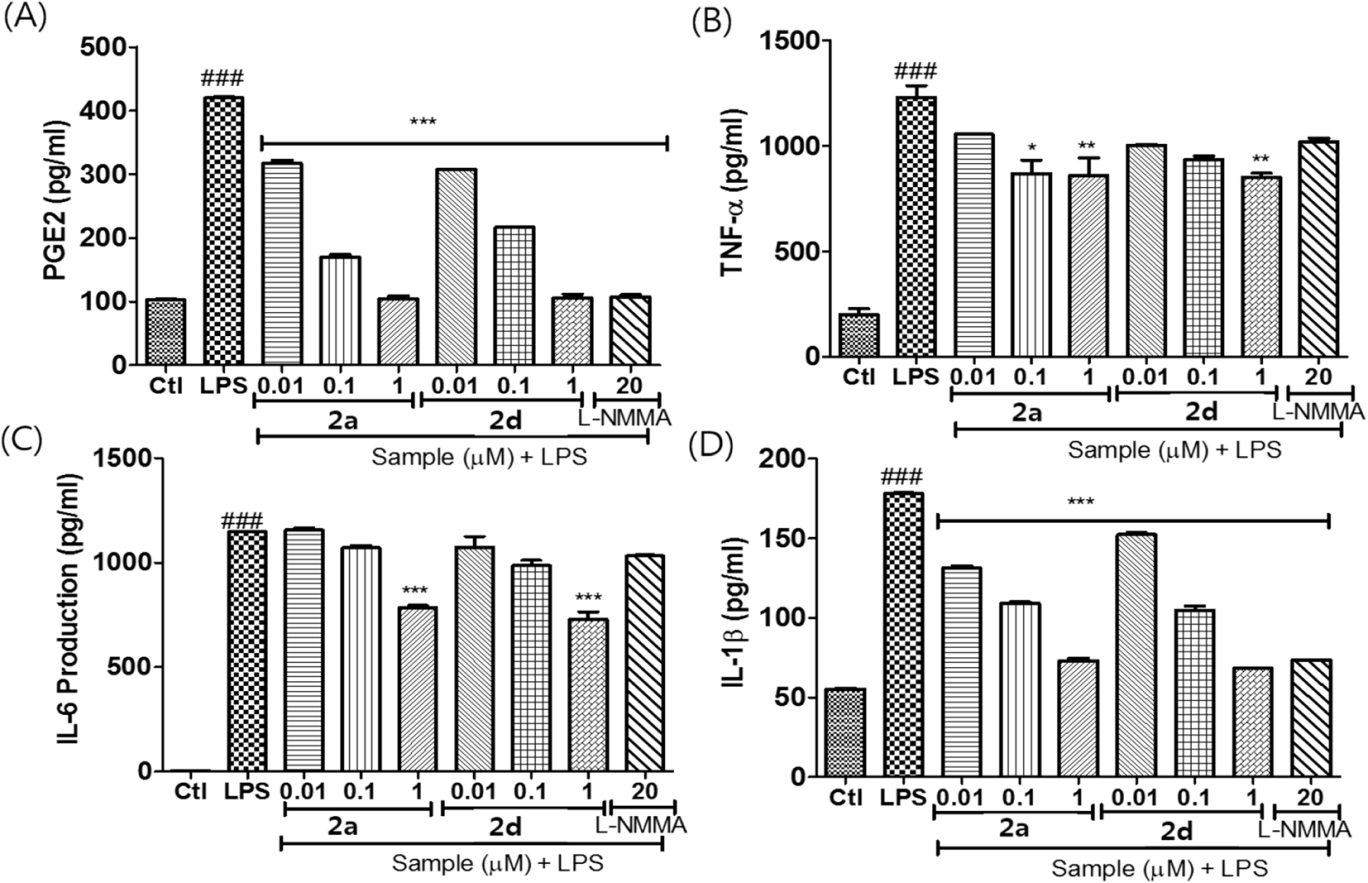
The control of LPS-induced inflammation by compounds **2a and 2d** in BV-2 cells: (**A**) the secreted level of PGE2 as a pro-inflammatory mediator, (**B**) TNF-α, (**C**) IL-6, and (**D**) IL-1β as pro-inflammatory cytokines.

The promising results of compounds **2a-r** encouraged us to check the regulation of pro-inflammatory cytokines and mediators related to NO production. Fortunately, the tested compounds regulated the expression levels of iNOS and COX-2. Compounds **2a** and **2d** (0.01 to 1 uM) showed efficacies similar to 20 uM level L-NMMA (Fig. 6). While compound **2a** showed limited potency and efficacy, compound **2d** downregulated iNOS and COX-2 more efficiently than compound **2a**, and the regulation of COX-2 showed concentration dependency. Compound **2d** presented 20-fold higher potency than L-NMMA, and the efficacy of the compound was retained at 0.01 µM. Finally, pro-inflammatory cytokines (TNF-α, IL-6, and IL-1β) and PGE2, another pro-inflammatory mediator, were studied with respect to compounds **2a** and **2d** (Fig. 7). The study also confirmed the superiority of compound **2d** with respect to compound **2a** or L-NMMA. Interestingly, compound **2d** also regulated the expression levels of PGE2, TNF-α, IL-6, and IL-1β in a concentration-dependent manner, confirmming their pharmacological potential on inflammation.

## Discussion and conclusions

### Kinase-like shape of 1,5-oxaza spiroquinone as an NE ring system

**Figure 8:**
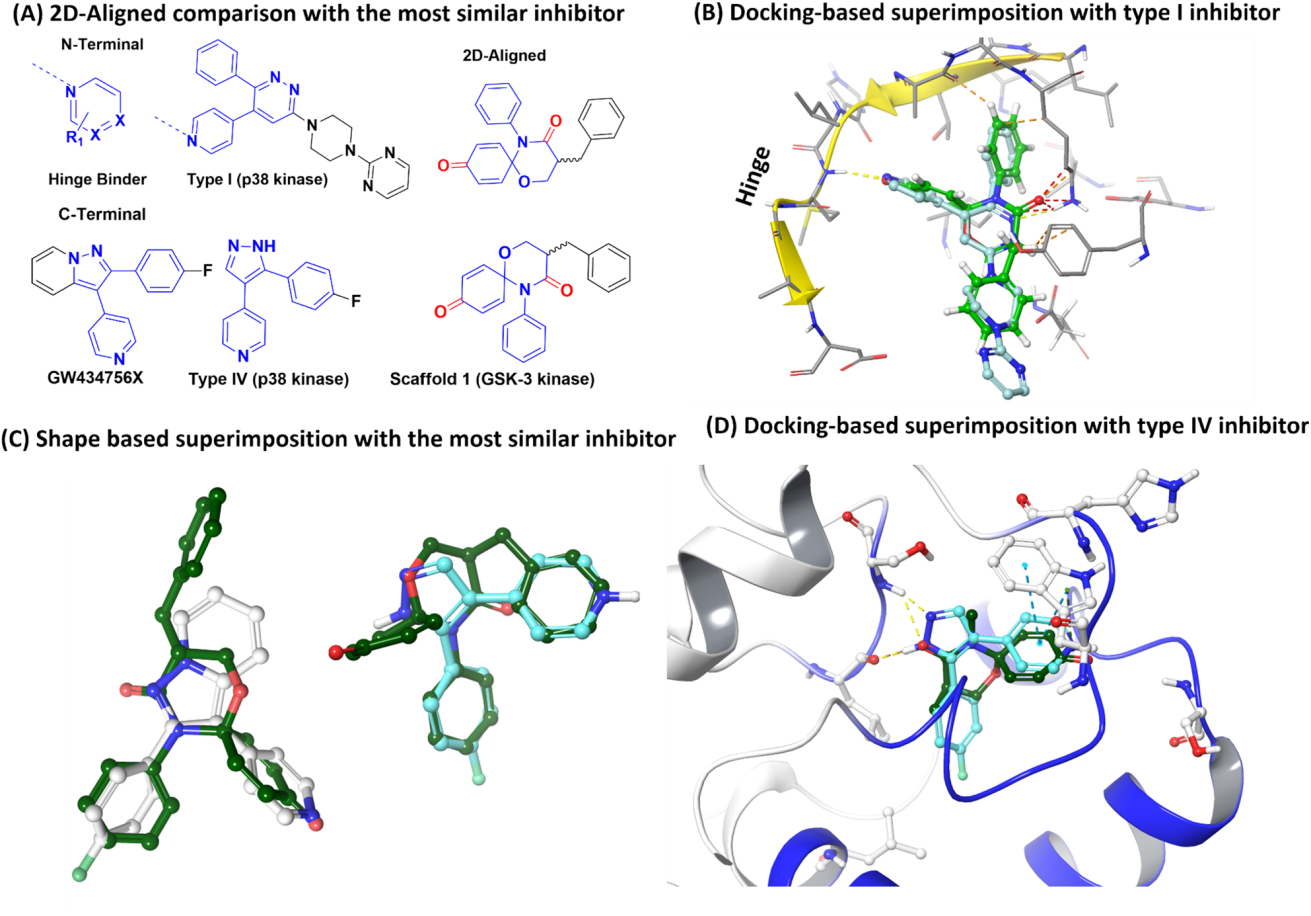
Molecular shape of 1,5-oxaza spiroquinone (**A**) 2D-aligned comparison of compound **1a** with the most similar kinase inhibitor, (**B**) docking-based superimposition of compound **1a** (green) with p38α type I inhibitor (PDB: 4EWQ), (**C**) 3D-shape based superimposition of compound **1a** (dark green) with the most similar kinase inhibitors (cyan: X-ray ligand of p38α (PDB: 3HVC), white: GW434756X), and (**D**) docking-based superimposition of compound **1a** without N-phenyl/α-benzyl substituents (dark green) with p38α type IV inhibitor (PDB: 3HVC). H-bond : dashed yellow, pi-pi stacking: dashed cyan, sterically poor: dashed orange, sterically forbidden: dashed red.

Ideally, if researchers can find unprecedented inhibitors in kinase space, the success rates of kinase therapies can be enhanced due to these enriched pools. In particular, biologically active ‘NE ring systems (not yet existing ring systems)’ can help researchers update the definition of what structures are kinase-like. NE ring systems can provide unique molecular shapes and pharmacophore features, different from known inhibitors. Although a pocket of a kinase cannot be occupied with any known inhibitor due to dissimilar shape or improper electrostatic property between the pocket and the inhibitor, NE ring systems are eligible for the pocket. Thus, unprecedented features of active NE ring systems can be useful for investigating novel binding modes (using known sites), as well as new binding sites of kinases. In the case of our spiro[5.5]undeca ring system, the core structure cannot exist within one flat space, and the quinone group and the oxaza ring are perpendicular to each other. The three rings of 1,5-oxazaquinone cannot exist on approximately one plane. In other words, one ring forces another ring out of the plane. At a glance, such a shape is very dissimilar to the shapes of common hinge binders, which are generally flat, so the NE ring system is not desirable for ATP competitive inhibition. If the spiroquinone core is close to a hinge residue to form a hydrogen bonding interaction, either the gatekeeper residue or the conserved Lys at the roof of the ATP binding site can exhibit steric hindrance via carbonyl groups (amide C=O or C=O of the quinone) or N-aryl groups, as seen in Fig. 8A–B. Otherwise, after the desirable positioning of the scaffold against the gatekeeper and P-loop, the scaffold cannot properly reach the hinge region. Therefore, the protruding carbonyl groups of this NE ring system can prevent the binding mode of type I kinase inhibitors to result in GSK-3 specificity. In addition, when we conducted a shape-based screening of the scaffold **1** with kinase inhibitors of ChEMBL DB according to our previous methods^9,51–53^, the most similar ring system with the scaffold **1** was 4,5-diaryl pyrazole motif (e.g., GW434756X, a p38 type IV inhibitor that is also active in GSK-3), as seen in Fig. 8C–D^54^. Notably, despite the 3D-similarity, it seems that differences in the pharmacophore vectors and the carbonyl groups protruding from the spiroquinone core cause the molecules to have dramatically different effects against p38 and GSK-3 (Fig. 8B). Consequently, despite the reduced efficiency of this NE ring system compared to kinase-like ring systems (Fig. 2A), it could provide a unique binding mode (Fig. 5) through modifying the known binding modes (Fig. 2).

### Molecular insight into the spiro[5.5]undeca ring system for targeting kinases

As a spiro[5.5]undeca ring system, our 1,5-oxaza spiroquinone did not satisfy the “2–0” rules, N(aromatic) + NH(aromatic) > 2 or Ar–NH + R-CN > 0^55^. Experimentally, compound **1c** also showed selective weak potency against 5 kinases (JNK1, CDK1/2, DAPK1, and PKCα) among the 379 protein kinases tested. After obtaining data on spiroquinone **1**, the optimization of the kinase-likeness of unprecedented scaffold **1** could be achieved by merging our scaffold **1** with a privileged Bemis Murcko framework: (1) an aniline (Ar−NH), (2) a bisarylaniline (Ar_1_−NH−Ar_2_), (3) a heteroaromatic group having nitrogens (N_aro_), and (4) a heteroaromatic group having NH (NH_aro_). Based on the efficiency of the synthetic route, R1 group modification can provide facile derivatization during the late-stage development of the scaffold **1** (Fig. 1). After the cyclization of the 1,5-oxaza ring, click chemistry for triazole formation (compounds **2a**-**r**) was the most atom- and step- economical method that did not interfere with other functionalities. The introduction of the triazole group is an example of a heteroaromatic group having nitrogens (N_aro_) to satisfy the “2–0” rules. As a result, dramatic changes in the kinase profiles were observed between JNK-1 and GSK-3. Notably, the potencies were also enhanced from compound **1c** to compound **2d**. During further SAR studies through the merging strategy, an introduction of a second privileged Bemis Murcko framework, such as a 3-pyridine group (**2b**), a cyano group (**2g**), and an acetamido group (**2l**), was considered. However, the introduction of this second group did not result in an impressive change in potency. The potencies of compounds **2j** and **2p,** as well as the specificity of compound **2d**, verified the kinase-like potential of scaffold **1**. To explore the cause of the selective bioactivity against GSK-3β, we performed docking followed by molecular dynamics to investigate the interaction pattern (Supplementary S3). At this moment, the origin of current improvement and the points for further improvements are not perfect. However, notably, our simulations indicate that the spiroquinone ring system could not perfectly occupy the ATP binding site of GSK-3 as do typical ATP competitive inhibitors. The molecular dynamics results of active compound **2j** or **2p** support that the key differential inhibitory effect of these compounds arises from hydrophobic interactions with the substrate binding pocket, particularly with residues Phe93/Phe67. Although most interactions are common for the main core of compounds **2**, the difference in the inhibitory activities can be explained by the residues surrounding the N-aryl substituent at the triazole ring. When a polar group is present near the triazole ring substituent (**2h**, **2l** and **2m**), the hydrophobicity of the 63 to 69 loop can decrease, supporting the decrease in inhibitory activity. The 3-benzylphenyl substituent (compound **2p**) could reach the Leu88 backbone to form a hydrogen bonding interaction as well as a π–π stacking interaction with Phe93/Phe67 to maximize the binding potential. The length of the substituent was also critical for reaching the edge of the 89 to 95 loop at site 2 and forming a hydrogen bond with the protein backbone, as well as a π–π stacking interaction with Phe93/Phe67. our simulations showed that a substituent on the triazole matches well with the protein if the abovementioned condition is satisfied. A comparison between the more and less active compounds showed differences in the synchronization through the RMSF values (Supplementary S4). In particular, the simulation results suggest that the ligand is shuffling between sites 1 and 2 and stabilized by the Lys85 anchor. The ligand movement towards site 2 provides an anchor for the water bridge interaction with Tyr134 instead of a hydrogen bonding and Phe67 interaction (Fig. 5A). The ligand forms a hydrogen bond with the phenolic OH group of Tyr134 (site 1) and then destabilizes the interaction with Phe67, which is compensated by the hydrophobic region of the 63 to 69 loop in site 2.

### Anti-inflammatory effect & target deconvolution

Well-known literature^56,57^ described that the inhibition of GSK-3 can result in the downregulation of microglial migration (activation) and the control of pro-inflammatory factors. Notably, GSK-3 has been proposed as a crucial regulator to balance pro- and anti-inflammatory cytokine production^56^. LPS is a known ligand of TLRs, a pathogen recognition receptor (PRR), so LPS binds to TLRs on the cell surface (including microglia) to induce inflammatory cytokines and nitric oxide production from NOS^3^. During this process, active GSK-3 can amplify the neuroinflammatory process^56,58^. In other words, induced pro-inflammatory cytokines were diminished by GSK-3 inhibitors (through the control of transcriptional factors) and NO production is also dependent on GSK-3^56–59^. On the other hand, the JNK signaling pathway mediates apoptosis and is critical for neuronal cytotoxicity, and JNK can be activated by nitric oxide stimulation^10^. Although studies on the role of GSK-3β in JNK activation are controversial, it was reported that GSK3 inhibition also promotes LPS-stimulated IFNb production via its ability to regulate c-Jun activity^60^. When integrating the literature related to JNK-1 and GSK-3, we expected that attenuated NO production by GSK-3 can decrease the activation of JNK-1. Therefore, it is expected that LPS-induced neuroinflammation can be better rescued by compound **2d** (GSK-3beta inhibitor with weak JNK1 inhibition) than by compound **1c** (weak JNK1 inhibitor). Unfortunately, the experimental correlation of the GSK-3β inhibitory activity with NO production assay was not linear. Contrary to our expectations, tested compounds **2a-r** showed similar NO inhibition levels (0.1 to 1 µM, as shown in Table 3). Further studies of compounds **2a** and **2d** on pro-inflammatory cytokines and mediators could be matched with regulation through GSK-3 inhibition. Basically, because increased NO production can be a reason for the overexpression of COX-2, the expression of COX-2 could also be regulated along with NO production, which is controlled by compound **2d**. Because lipid mediators, such as PGE2, are generated from arachidonic acid by COX and PGE synthases, the inhibition of PGE2 production was expected and was observed with pro-inflammatory cytokines (TNF-α, IL-6, and IL-1β). However, this experimental evidence cannot exclude another target molecule of compounds **2a-r**. The current results suggest further molecular studies for deciphering the current anti-inflammatory efficacies of the compounds **2a-r**. Current study of compounds **1c** and **2d** also encourage us to further study the polypharmacology of the spiro[5.5]undeca scaffold to investigate its clinical positioning in the near future.

## Experimental section

### Chemistry

Every general chemistry procedure of this study is identical to our previous study on the scaffold^10^. All of the reagents and solvents were used without further purification. General reactions were performed under an atmosphere nitrogen with magnetically stirring and reaction monitoring was conducted by analytical thin layer chromatography (TLC) in the visualized condition of UV light (254 nm) and staining (Ninhydrine & PMA solutions) with heating. Flash column chromatography was performed on silica gel (230–400 mesh). ^1^H and ^13^C NMR spectra were analyzed using Brucker 600 MHz and chemical shifts were quoted in parts per million (ppm) with the calibration through the residual proton and carbon resonance of the solvent (CDCl_3_ δH 7.25, δC 77.0; MeOD δH 3.31, δC 49.15). The spectra data was reported as follows: s = singlet, d = doublet, t = triplet, q = quartet, m = multiplet, dd = doublet of doublet, ddd = doublet of doublet of doublet, br = broad signal. J, were reported in hertz unit (Hz). ^13^C NMR was fully decoupled by broad band proton decoupling. Mass spectral data were obtained under the condition of Agilent LC/Q-TOP by using ESI positive method.
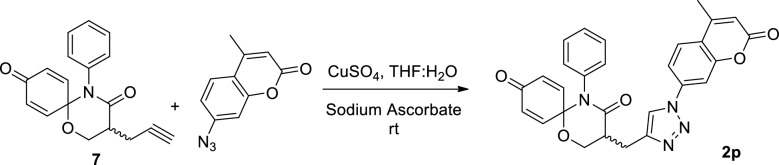


To a solution of compound **7** (1 eq.) in 1:1 THF and H_2_O mixture, aromatic azide (2 eq.) was added, and then followed by addition of CuSO4 (2 eq.) and sodium acerbate (1 eq.) at room temperature. The reaction mixture was stirred 12–15 h and reaction was monitored by TLC. After completion of reaction, the reaction mixture was filtered through celite. The filtrate was washed with water. The organic layer was washed with brine solution and organic layers was dried over sodium sulphate and evaporated under reduced pressure to afford the crude residue, which was purified by silica gel column chromatography by eluting with ethyl acetate/hexane to yield corresponding triazole derivatives. All synthesized triazole derivatives were confirmed by the spectral analysis. Brown gummy liquid; 85% yield; IR (neat): 2988, 2862, 2718, 1740, 1710, 1689, 1489, 1270, 1098, 733 cm^−1^; ^1^H NMR (600 MHz, CDCl_3_) δ 7.96 (s, 1H), 7.78–7.73 (m, 2H), 7.71 (d, *J* = 1.1 Hz, 1H), 7.33–7.27 (m, 3H), 7.19 (dd, *J* = 10.3, 3.2 Hz, 1H), 7.02–6.96 (m, 2H), 6.83 (dd, *J* = 10.2, 3.2 Hz, 1H), 6.37 (d, *J* = 1.1 Hz, 1H), 6.12 (dd, *J* = 10.3, 2.0 Hz, 1H), 6.03 (dd, *J* = 10.2, 2.0 Hz, 1H), 4.62 (dd, *J* = 12.1, 9.5 Hz, 1H), 4.40 (dd, *J* = 12.2, 6.4 Hz, 1H), 3.43 (dd, *J* = 15.0, 6.8 Hz, 1H), 3.38–3.32 (m, 1H), 3.20 (dd, *J* = 15.0, 3.9 Hz, 1H), 2.49 (d, *J* = 1.1 Hz, 3H); ^13^C NMR (150 MHz, CDCl_3_) δ 184.00, 169.59, 159.96, 151.51, 144.68, 142.89, 136.41, 133.40, 129.94, 129.90, 129.81 (3 C), 129.17 (4 C), 129.11, 126.29, 120.81, 115.78, 115.74, 108.40, 83.53, 63.80, 41.51, 23.67, 18.76 ppm; HRMS (EI, m/z): M^+^ calculated for C_28_H_22_N_4_O_5_ 494.1590, Found 494.1575.

### Molecular docking simulations

As the best PDB, 1J1B was selected for sites 1 and 7 and 3I4B was chosen for sites 2 and 5, in addition to the merged model of sites 1 and 2. Furthermore, 1Q3W was selected for site 4 and 1Q4L for site 6 modeling. The reliability of the docking protocol for GSK3 was validated through redocking of respective binding modes (RMSD between redocking pose and crystal pose: BDBM8269-ligand of 1Q4L = 0.6475 Å, BDBM35641-ligand of 3I4B = 0.8745 Å, see also supplementary S2). The ligands were sketched and subjected to ligand preparation using the LigPrep module of the Schrodinger software suite (2018). The resulting ligands are subjected to ConfGen for generating 100 minimized conformations for each ligand for preparation of molecular docking. Structures of GSK-3 were retrieved from the PDB with the identification numbers 2j2b, 3I4B and 1Q4L. The initial structure of the protein was refined and subjected to energy minimization in order to ensure correct starting structures. All heteroatoms (except inhibitor and metal ion) were removed from protein files. All water molecules (beyond 3 Å from the inhibitor) and the rest of the chains (except A) were removed from the complex, and the protein was minimized using the OPLS3e force field. Furthermore, H atoms were added to the protein to correct ionization and tautomeric states of amino acid residues. A receptor grid was generated with a 30 Å size for each site by selecting centroid amino acids using receptor grid generation in the Glide application based on published probable sites (7 sites). The ligands were docked to the protein using the extra precision mode (XP) Glide docking protocol after respective receptor grid generation. Ten poses were written for each ligand. OPLS3e was used to calculate the binding energy and protein–ligand binding poses for each compound. All of the docking poses of selected ligands at six binding sites were collected, sorted by docking score, and filtered by top 1000 scores. Docking scores of two different sites could not be normalized due to insufficient data for describing the probability density function of the docking score in each site. The percent probability was calculated by shorting the ligand based on the docking score after clubbing all selected receptor results.

### Molecular dynamics simulation

The MD calculations were performed with the help of Desmond to evaluate the protein–ligand interactions in solvated conditions. The modeled protein–ligand complexes were embedded through docking in the TIP3P water model. Orthorhombic periodic boundary conditions were selected to specify the shape and size of the repeating unit with minimization of the total volume. The protein–ligand complex system was neutralized with the counterions, and a physiological salt concentration was used at 0.15 M. The system was developed and minimized with default settings (except time and temperature). The relaxed system was subjected to 50 ns simulation time with a time step of 2 fs in the constant number, pressure, and temperature (NPT) ensemble using a Nose–Hoover thermostat at 310 K and Martyna–Tobias–Klein barostats at 1.01 bar pressure. Every trajectory was recorded with a time interval of 20 ps. Energy potential, root mean square deviation (RMSD), root mean square fluctuations (RMSF) and intermolecular hydrogen bond interactions were monitored for the stability of the protein–ligand complex and analyzed to understand the underpinning of the dynamic interaction. The MD simulations of the chosen docking complex (site 1 and sites 1/2) were conducted to sample all possible scenarios of the interaction in dynamic stages of the enzyme and to validate the above binding mode of molecular docking.

### Reagents and cell culture

Lipopolysaccharide (LPS), were obtained from Sigma chemical (St. Louis, MO). Penicillin–streptomycin (PS), Fetal bovine serum (FBS), Dulbecco modified Eagle medium (DMEM) were brought from Invitrogen (Carlsbad, CA, USA). sulfanilamide and 0.1% N-1-napthylethylenediamine dihydrochloride were purchased from sigma chemicals. Prostaglandin E2 (PGE2), Interleukin-6 (IL-6), tumor necrosis factor alpha (TNF-α) and interleukin (IL-1β) elisa kit was purchased from R&D Systems (Minneapolis, MN, USA). Primary antibodies against various proteins such as inducible nitric oxide synthase (iNOS), cyclooxygenase 2 (COX-2), and α-tubulin were obtained from abcam, Santa Cruz technology and cell signaling technology. Secondary antibodies against rabbit, goat and mouse were obtained from sigma chemicals. Murine microglial cell BV2 were used to evaluate the effect of compound against LPS activated microglia. BV2 cell we obtained as a gift sample by Dr. E Choi from Korea University, Seoul South Korea. This cell was maintained with high glucose DMEM supplemented with10% heat-inactivated FBS and 1% of mixture of penicillin–streptomycin (penicillin (1 × 10^5^ U/L), and streptomycin (100 mg/L)). Cultured cells were maintained by keeping the in a humidified incubator with 5% CO_2_ at 37 °C.

### Kinase assays

Every inhibitory activity of tested compounds was measured by radiolabelled method of Reaction Biology Corp). The radiolabelled ATP ([γ-^33^P] ATP) replaced a substrate with ^33^P-phosphorylated substrate so that the activity of a kinase was measured from the radiolabelled phosphorylated substrate. Kinase panel assay was tested at 30 μM testing compounds with an ATP concentration of 10 μM and a substrate concentration of 10 μM. % Residual activity against GSK3β was measured at 10 uM compounds **1** and **2**. ATP competitive assayof compound **2j** was measured in 10-dose IC_50_ mode with 3 or fourfold serial dilution starting at 10 μM. Curve fits of control compounds were performed where the enzyme activities at the highest concentration of compounds were less than 65%. The concentration of DMSO was controlled and raw data, % kinase activity was calculated relative to DMSO controls.

### Cell treatment, nitric oxide and cell viability assay

3-(4,5-dimethylthiazol-2-yl)-2,5-diphenyl-tetrazolium bromide (MTT) assay was performed to evaluate the cytotoxicity of the samples to the microglia. BV2 cells at the density of 4 × 10^4^ cells /well were seeded in the 96 well plate and incubated for 24 h. Seeded cells were pre-treated with different concentration of the samples, and LPS (100 ng/ml) activation was performed after 30 min of compound treatment. Treated plate were incubated for 24 h in the incubator and nitric oxide assay and cell viability assay was performed. Treated cell’s conditioned medium was used to evaluate the NO assay, and the attached cells were used to evaluate the cell viability assay. 50 µL of conditioned medium (CM) from treated cells were transferred to a new 96 well plate for NO assay. This CM was mixed with the equal volume of the mixture of Gries reagent ((1% sulfanilamide and 0.1% N-1-napthylethylenediamine dihydrochloride). NaNO2 was used as standard and the colorimetric change were evidenced by measuring its OD at 540 nm in a microplate reader. The cells in the plate were incubated for 1 h with MTT solution making it the final concentration of 0.5 µg/ml. Blue stained cells were incubated with 200 ul of DMSO and the solution get changed to purple/formazan color and it was observed by measuring the absorbance at 570 nm in a microplate reader.NG-mono-methyl-L-arginine (L-NMMA), a well-known nitric oxide synthase (NOS) inhibitor, was used as a positive control.

### Measurement of PGE2, TNF-α, IL-1β, and IL-6 production

To evaluate the secreted level of Prostaglandin E2 (PGE2), Tumor necrosis factor (TNF-α), Interleukin (IL-6), Interleukin (IL-1β), BV2 cells were seeded in a 6 well plate at the density of 1.5 × 10^6^ cells/well in DMEM and incubated for 24 h. Seeded cells sere pretreated and, activated with LPS for next 24 h. conditioned medium from treated cells were collected and stored at -80 degree for longer period of time. The amount of TNF-α, IL-1β, PGE2,and IL-6 were evaluated using the competitive enzyme immunoassay kit. PGE2 kit were obtained from (Cayman Chemical, Ann Arbor, MI, USA) and TNF-α, IL-1β, and IL-6 were measured using ELISA development kits (R&D Systems, Minneapolis, MN, USA). All the protocol was exactly followed as written in the manual.

### Western blot analysis

Western blot analysis was performed to see the protein expression of the iNOS and COX-2 in the BV2 cells. BV2 cells were seeded in a 6 well plate at the density of 6 × 10^5^ cells/well overnight and cells were treated as mentioned earlier in the cell treatment section and this time treatment was performed for 6 h only. After 6 h, cells were harvested and lysed with lysis buffer containing (RIPA, protease inhibitor & phosphatase inhibitor), protein estimation was performed using biorad assay and the 30 µg of total protein was separated by 8% SDS-PAGE gel electrophoresis, transferred to nitrocellulose membranes, and incubated with primary antibodies against tubulin, iNOS, COX-2, and α-tubulin. PVDF membranes were incubated with horseradish peroxidase-conjugated secondary antibodies, and protein bands were visualized using ECL Western Blotting Detection Reagents (Amersham Pharmacia Biotech). Quantitative analysis of the protein bands was quantified using Image Master 2D Elite software (version 3.1, Amersham Pharmacia Biotech).

### Statistical analysis

All results are expressed as mean ± standard error of the mean (SEM). Statistical significance between experimental groups were determined by using one-way analysis of variance (ANOVA) followed by the Tukey post-hoc test using GraphPad Prism 5 (GraphPad Software Inc., La Jolla, CA, USA). Statistical significance was set at *P* < 0.05. Each experiment was performed in triplicate.

## Supplementary information

Supplementary Information.
