# Supplementary material for: Positioning of an unprecedented spiro[5.5]undeca ring system into kinase inhibitor space
Source: Sci Rep. 2020 Dec 4;10:21265. doi: 10.1038/s41598-020-78158-9 (PMC7719162; doi:10.1038/s41598-020-78158-9)
Supplement: Supplementary file 1 — Supplementary Information. [file 41598_2020_78158_MOESM1_ESM.docx]

**Supporting Information**

**Positioning of an unprecedented spiro[5.5]undeca ring system into kinase inhibitor space**

ArramshettiVenkanna^a,†^, LalitaSubedi^a,†^, Mahesh K. Teli^a,†^, PremaDhorma Lama^a^, Bhargav Gupta Nangunuri, Sang-Yoon Lee^b^,Sun Yeou Kim^a,^* and Mi-hyun Kim^a,^*

*^a^Gachon Institute of Pharmaceutical Science and Department of Pharmacy, College of Pharmacy, Gachon University, Yeonsu-gu, Incheon, Republic of Korea.^b^Gachon Advanced Institute for Health Science and Technology, Graduate School and Neuroscience Research Institute, Gachon University, Yeonsu-gu, Incheon, Republic of Korea*

^†^*These authors are co-first authors.*

* Authors for correspondence e-mail: [kmh0515@gachon.ac.kr](mailto:kmh0515@gachon.ac.kr);[sunnykim@gachon.ac.kr](mailto:sunnykim@gachon.ac.kr)

**Table of contents**

| **File No.** | **Content** | **Page No.** |
| --- | --- | --- |
| **S1** | Synthetic procedure &identification of products | **2-18** |
| **S2** | Redocking experiment to validate the performance of Docking program | **19** |
| **S3** | Selected active inhibitors interaction with GSK3beta residue during MD simulation | **20** |
| **S4** | Figure representing the significance of contribution and interaction pattern at triazole substitution for active vs inactive | **21** |
| **S5** | Representation of the RMSD of protein and respective ligand during the molecular dynamics simulation | **22** |
| **S6** | ADMET parameter of the synthesized compounds | **23-24** |
| **S7** | Kinase panel screening of compound **2d** | **25-26** |
| **S8** | NO production assay & cell viability of chosen compounds | **27-28** |
| **S9** | Original blots of compounds **2a** and **2d** |  |
| **S10** | 1H and 13C NMR spectra | **28-50** |

**Supplementary File S1:** Synthetic procedure&identification of products

**Preparation of Azide partners 7a-r**

1. **Preparation of Azide partners:**

Different substituted Anilines (1 eq.) wasdissolved in HCl (6M) then the mixture was cooled to 0°C. A solution of NaNO_2_(1.5 equiv) in waterwas added slowly drop wise with a dropping funnel and the reaction mixture was allowed tostir for 30 min at 0°C.After stirring 30 min at 0°C**,** a solution of NaN_3_(4 eq.) in water wasadded dropwise at same temperature and slowly allowed to room temperature, and stirring was allowed for 1.5 h. The crude reaction mixture was extracted with Ethyl acetate, the organic layer was washed with water 2 times The organic layer was dried using anhydrous Na_2_SO_4_ and concentrated reduced pressure, and the crude product was subjected to flash column chromatography (ethyl acetate-hexane) to give corresponding azides.

**Note:** 1. Rota temperature does not exceed 15^o^C.

**General Experimental Preparation of different triazole derivatives**

To a solution of Spiro alkynes compound (1 eq.) in1:1 THF and H_2_O mixture, Aromatic azide (2 eq.) was added then followed by addition of CuSO4 (2 eq.) and Sodium Acerbate (1 eq.) at room temperature. The reaction mixture was stirred 12-15 h and reaction was monitored by TLC. After completion of reaction, the reactionmixture was filtered through celite. The filtrate was washed with water. The organic layer was washed with brine solution and organic layers was dried over sodium sulphate and evaporated under reducedpressure to afford the crude residue, whichwas purified by silica gel column chromatographyby eluting with ethyl acetate/hexane toyield corresponding triazole derivatives. All synthesized triazole derivatives were confirmed by thespectral analysis (FTIR, 1H NMR, 13C NMR and Mass spectroscopy).

| **Entry** | **Azide partner** | **Time**  **(hr)** | **Yield (%)** | **Entry** | **Azide partner** | **Time**  **(hr)** | **Yield (%)** |
| --- | --- | --- | --- | --- | --- | --- | --- |
| 2a |  | 10 | 94 | 2j |  | 11.5 | 84 |
| 2b |  | 12 | 92 | 2k |  | 12 | 80 |
| 2c |  | 10.5 | 88 | 2l |  | 10 | 78 |
| 2d |  | 10 | 90 | 2m |  | 8 | 74 |
| 2e |  | 9 | 95 | 2n |  | 9 | 76 |
| 2f |  | 11 | 89 | 2o |  | 10 | 96 |
| 2g |  | 12 | 87 | 2p |  | 11.5 | 85 |
| 2h |  | 8.5 | 93 | 2q |  | 13 | 89 |
| 2i |  | 9 | 92 | 2r |  | 9.5 | 82 |

**Methyl 2-((4-(methoxymethoxy)phenyl)(phenyl)carbamoyl)pent-4-ynoate (4)**

Light brown liquid, 92% yield; IR (neat): 3200, 2798, 2875, 2930, 1710, 1680, 1571, 1364, 1028, 862, 715 cm^-1^,^1^H NMR (600 MHz, CDCl_3_) δ 7.45 – 7.38 (m, 1H), 7.37 – 7.27 (m, 4H), 7.25 – 7.16 (m, 2H), 7.07 (d, *J* = 8.6 Hz, 1H), 6.99 (dd, *J* = 8.1, 5.7 Hz, 1H), 5.24 – 5.06 (m, 2H), 3.94 – 3.79 (m, 1H), 3.73 (s, 2H), 3.64 (s, 1H), 3.54 – 3.38 (m, 3H), 3.05 – 2.89 (m, 1H), 2.85 (dd, *J* = 17.5, 2.4 Hz, 1/2H), 2.80 – 2.67 (m, 1/2H), 2.09 (t, *J* = 2.7 Hz, 1/2H), 2.07 (t, *J* = 2.7 Hz, 1/2H); ^13^C NMR (150 MHz, CDCl_3_) δ 169.95, 168.95, 157.07, 142.56, 136.44, 136.03, 130.36, 129.89, 128.98, 128.27, 127.70, 126.32, 117.27, 116.67, 94.51, 80.88, 70.36, 57.09, 52.78, 48.22, 19.15 ppm; HRMS (EI, m/z): [M+H]+ calculated for C_21_H_21_NO_5_ 367.1420, Found367.1412.

**2-(Hydroxymethyl)-N-(4-(methoxymethoxy) phenyl)-N-phenylpent-4-ynamide (5):**

White gummy liquid, 82% yield; IR (neat): bs 3500, 3230, 2798, 2875, 2930, 1715, 1658, 1570, 1376, 1280, 1097, 852, 689 cm^-1^,^1^H NMR (600 MHz, CDCl_3_) δ 7.45 – 7.40 (m, 1H), 7.32-7.29 (m 2H), 7.27 (d, *J* = 5.0 Hz, 2H), 7.19 (d, *J* = 8.4 Hz, 2H), 7.07 (d, *J* = 8.1 Hz, 1H), 7.00 (d, *J* = 8.0 Hz, 1H), 5.16 (d, *J* = 29.3 Hz, 2H), 3.82 (t, *J* = 5.2 Hz, 2H), 3.47 (d, *J* = 30.0 Hz, 3H), 3.08-2.98 (m, 1H), 2.82 – 2.72 (m, 1H), 2.63 (dd, *J* = 15.9, 6.8 Hz, 1H), 2.44 (dd, *J* = 16.7, 6.3 Hz, 1H); ^13^C NMR (150 MHz, CDCl_3_) δ 174.20, 156.74, 142.35, 136.16, 135.81, 129.80, 128.85, 128.51, 128.40, 127.63, 126.27, 117.15, 116.53, 94.32, 81.14, 70.11, 63.02, 56.09, 42.69, 18.67 ppm; HRMS (EI, m/z): [M+H]+ calculated for C_21_H_21_NO_5_ 367.1420, Found367.1412.

**2-(Hydroxymethyl)-N-(4-hydroxyphenyl)-N-phenylpent-4-ynamide (6):**

White solid; 88% yield; M.P.:148.8-152.6^o^C; IR (neat): bs 3600, 3360, 2970, 2918, 2768, 1710, 1640, 1582, 1518, 1268, 1226, 868, 715 cm^-1^;^1^H NMR (600 MHz, MeOD) δ 7.47 – 7.40 (m, 1H), 7.37 – 7.23 (m, 4H), 7.20 (t, *J* = 7.2 Hz, 1H), 7.12 (d, *J* = 8.5 Hz, 1H), 6.84 (d, *J* = 8.6 Hz, 1H), 6.76 (d, *J* = 8.5 Hz, 1H), 3.78 (dd, *J* = 10.6, 7.7 Hz, 1H), 3.56 (dd, *J* = 10.7, 5.9 Hz, 1H), 3.14 – 3.00 (m, 1H), 2.45 (dd, *J* = 16.5, 9.1 Hz, 1H), 2.41 (s, 1H), 2.37 – 2.29 (m, 1H);^13^C NMR (150 MHz, MeOD) δ 174.41, 157.25, 143.18, 134.61, 133.97, 129.96, 129.34, 128.60, 127.94, 126.53, 126.23, 115.80, 115.24, 80.96, 69.91, 62.72, 44.53, 18.14 ppm;HRMS (EI, m/z): [M+H]+ calculated for C_18_H_17_NO_3_295.1208, Found 295.1207.

**5-Phenyl-3-(prop-2-yn-1-yl)-1-oxa-5-azaspiro[5.5]undeca-7,10-diene-4,9-dione (7):**

White liquid; 82% yield; purity 98.4%; IR (neat):3340, 2978, 2960, 2738, 1708, 1680, 1581, 1548, 1468, 1258, 868, 715 cm^-1^;^1^H NMR (600 MHz, CDCl_3_) δ 7.35 – 7.32 (m, 3H), 7.17 (dd, *J* = 10.3, 3.2 Hz, 1H), 7.10 – 7.05 (m, 2H), 6.91 (dd, *J* = 10.2, 3.2 Hz, 1H), 6.16 (dd, *J* = 10.3, 2.0 Hz, 1H), 6.08 (dd, *J* = 10.2, 2.0 Hz, 1H), 4.42 (d, *J* = 7.5 Hz, 2H), 3.07 – 3.00 (m, 1H), 2.83 (ddd, *J* = 17.0, 8.3, 2.6 Hz, 1H), 2.75 (ddd, *J* = 17.0, 4.2, 2.7 Hz, 1H), 2.15 (t, *J* = 2.7 Hz, 1H); ^13^C NMR (150 MHz, CDCl_3_) δ 183.73, 168.37, 144.06, 142.92, 136.14, 129.74 (2 C), 129.64 (2 C), 128.96 (2 C), 128.90, 83.24, 80.31, 70.85, 63.36, 40.21, 17.91 ppm; HRMS (EI, m/z): [M+H]+ calculated for C_18_H_15_NO_3_293.1052, Found 293.1069.

**5-Phenyl-3-((1-phenyl-1H-1,2,3-triazol-4-yl)methyl)-1-oxa-5-azaspiro[5.5]undeca-7,10-diene-4,9-dione (2a):**

Light brown liquid; 94% yield; purity 97.8%; IR (neat): 2978, 2940, 2856, 2758, 1710, 1686, 1538, 1518, 1489, 1358, 1260, 868, 705 cm^-1^;^1^H NMR (600 MHz, CDCl_3_) δ 7.89 (s, 1H), 7.74 (dd, *J* = 8.6, 1.1 Hz, 2H), 7.55 (t, *J* = 7.9 Hz, 2H), 7.49 – 7.45 (m, 1H), 7.33 – 7.26 (m, 3H), 7.18 (dd, *J* = 10.3, 3.2 Hz, 1H), 7.00 – 6.97 (m, 2H), 6.84 (dd, *J* = 10.2, 3.2 Hz, 1H), 6.12 (dd, *J* = 10.3, 2.0 Hz, 1H), 6.03 (dd, *J* = 10.2, 2.0 Hz, 1H), 4.65 (dd, *J* = 12.2, 9.6 Hz, 1H), 4.40 (dd, *J* = 12.2, 6.6 Hz, 1H), 3.45 (dd, *J* = 15.0, 6.9 Hz, 1H), 3.41 – 3.31 (m, 1H), 3.19 (dd, *J* = 15.0, 3.8 Hz, 1H); ^13^C NMR (150 MHz, CDCl_3_) δ 184.02, 169.61, 144.74, 142.93, 136.97, 136.43, 129.84, 129.81 (4 C), 129.08 (3 C), 128.99, 128.80 (2 C), 121.00, 120.40 (2 C), 83.44, 63.71, 41.55, 23.65 ppm;HRMS (EI, m/z): [M+H]+ calculated for C_24_H_20_N_4_O_3_412.1535, Found 412.1530.

**5-Phenyl-3-((1-(pyridin-3-yl)-1H-1,2,3-triazol-4-yl)methyl)-1-oxa-5-azaspiro[5.5]undeca-7,10-diene-4,9-dione (2b):**

Dark brown liquid; 92% yield; purity 98.9%; IR (neat): 2972, 2854, 2760, 1708, 1697 1619,, 1535, 1479, 1260, 867, 715 cm^-1^;^1^H NMR (600 MHz, CDCl_3_) δ 8.12 (d, *J* = 8.1 Hz, 1H), 7.94 (s, 1H), 7.51 (dd, *J* = 24.5, 6.8 Hz, 2H), 7.28 (dd, *J* = 4.8, 2.5 Hz, 4H), 7.19 (dd, *J* = 10.3, 3.2 Hz, 1H), 7.01 – 6.96 (m, 2H), 6.83 (dd, *J* = 10.2, 3.2 Hz, 1H), 6.12 (dd, *J* = 10.3, 2.0 Hz, 1H), 6.03 (dd, *J* = 10.2, 2.0 Hz, 1H), 4.61 (dd, *J* = 12.1, 9.6 Hz, 1H), 4.40 (dd, *J* = 12.2, 6.4 Hz, 1H), 3.43 (dd, *J* = 15.0, 6.8 Hz, 1H), 3.38 – 3.32 (m, 1H), 3.20 (dd, *J* = 15.0, 4.0 Hz, 1H); ^13^C NMR (150 MHz, CDCl_3_) δ 183.87, 169.46, 144.56, 142.76, 136.28, 129.80 (2 C), 129.77 (2 C), 129.67 (2 C), 129.03 (3 C), 128.97, 128.89, 127.79, 120.80, 119.83, 83.40, 63.65, 41.41, 23.53 ppm;HRMS (EI, m/z): [M+H]+ calculated for C_23_H_19_N_5_O_3_413.1488, Found 413.1482.

**3-((1-(4-Fluorophenyl)-1H-1,2,3-triazol-4-yl)methyl)-5-phenyl-1-oxa-5-azaspiro[5.5]undeca-7,10-diene-4,9-dione(2c):**

Yellow liquid; 88% yield; purity 98.4%; IR (neat): 2980, 2935, 2719, 1720, 1673 1640, 1538, 1261, 882, 705 cm^-1^;^1^H NMR (600 MHz, CDCl_3_) δ 7.85 (s, 1H), 7.75 – 7.69 (m, 2H), 7.34 – 7.27 (m, 3H), 7.27 – 7.22 (m, 2H), 7.20 (dd, *J* = 10.3, 3.2 Hz, 1H), 6.98 (d, *J* = 6.3 Hz, 2H), 6.84 (dd, *J* = 10.2, 3.2 Hz, 1H), 6.13 (dd, *J* = 10.3, 2.0 Hz, 1H), 6.03 (dd, *J* = 10.2, 2.0 Hz, 1H), 4.65 (dd, *J* = 12.1, 9.6 Hz, 1H), 4.40 (dd, *J* = 12.2, 6.5 Hz, 1H), 3.43 (dd, *J* = 15.0, 6.9 Hz, 1H), 3.39 – 3.33 (m, 1H), 3.19 (dd, *J* = 14.9, 3.8 Hz, 1H); ^13^C NMR (150 MHz, CDCl_3_) δ 183.76, 169.34, 162.19 (d, *J* = 249.3 Hz, C-F), 145.01, 144.49, 142.67, 136.20, 133.02, 129.63, 129.60, 129.56, 128.86 (2 C), 128.79, 122.16, 122.10, 120.91, 116.63, 116.47, 83.23, 63.52, 41.31, 23.40 ppm;HRMS (EI, m/z): [M+H]+ calculated for C_24_H_19_FN_4_O_3_430.1441, Found 430.1430.

**3-((1-(3-Chlorophenyl)-1H-1,2,3-triazol-4-yl)methyl)-5-phenyl-1-oxa-5-azaspiro[5.5]undeca-7,10-diene-4,9-dione (2d):**

Light yellow liquid; 90% yield; purity 98.9%; IR (neat): 2930, 2926, 2836, 2738, 1738, 1671 1646, 1478, 715 cm^-1^;^1^H NMR (600 MHz, CDCl_3_) δ 7.90 (s, 1H), 7.80 (t, *J* = 2.0 Hz, 1H), 7.64 (ddd, *J* = 8.0, 1.9, 1.0 Hz, 1H), 7.53 – 7.42 (m, 2H), 7.35 – 7.28 (m, 3H), 7.17 (dd, *J* = 10.3, 3.2 Hz, 1H), 7.03 – 6.94 (m, 2H), 6.84 (dd, *J* = 10.2, 3.2 Hz, 1H), 6.13 (dd, *J* = 10.3, 2.0 Hz, 1H), 6.04 (dd, *J* = 10.2, 2.0 Hz, 1H), 4.62 (dd, *J* = 12.2, 9.6 Hz, 1H), 4.40 (dd, *J* = 12.2, 6.5 Hz, 1H), 3.44 (dd, *J* = 15.0, 6.8 Hz, 1H), 3.38 – 3.30 (m, 1H), 3.19 (dd, *J* = 15.0, 3.9 Hz, 1H); ^13^C NMR (150 MHz, CDCl_3_) δ 183.96, 169.56, 145.37, 144.68, 142.82, 137.77, 136.37, 135.65, 130.87, 129.86, 129.84, 129.77 (2 C), 129.10 (2 C), 129.04, 128.83, 120.91, 120.64, 118.29, 83.45, 63.68, 41.51, 23.62 ppm; HRMS (EI, m/z): [M+H]+ calculated for C_24_H_19_ClN_4_O_3_446.1146, Found 446.1149.

**3-((1-(4-Bromophenyl)-1H-1,2,3-triazol-4-yl)methyl)-5-phenyl-1-oxa-5-azaspiro[5.5]undeca-7,10-diene-4,9-dione(2e):**

Light brown liquid; 95% yield; purity 98.4%; IR (neat): 2932, 2864, 2736, 1728, 1680, 1578, 1439, 705 cm^-1^;^1^H NMR (600 MHz, CDCl_3_) δ 7.88 (s, 1H), 7.71 – 7.66 (m, 2H), 7.66 – 7.61 (m, 2H), 7.28-7.28 (m, 3H), 7.18 (dd, *J* = 10.3, 3.2 Hz, 1H), 6.98 (d, *J* = 6.4 Hz, 2H), 6.84 (dd, *J* = 10.2, 3.2 Hz, 1H), 6.13 (dd, *J* = 10.3, 2.0 Hz, 1H), 6.04 (dd, *J* = 10.2, 2.0 Hz, 1H), 4.64 (dd, *J* = 12.1, 9.7 Hz, 1H), 4.40 (dd, *J* = 12.2, 6.5 Hz, 1H), 3.43 (dd, *J* = 15.0, 6.8 Hz, 1H), 3.39 – 3.32 (m, 1H), 3.18 (dd, *J* = 15.0, 3.9 Hz, 1H); ^13^C NMR (150 MHz, CDCl_3_) δ 183.95, 169.54, 144.68, 142.83, 136.39, 135.91, 132.94, 129.86, 129.82, 129.76 (3 C), 129.09 (4 C), 129.02, 122.40, 121.73, 120.79, 83.45, 63.71, 41.52, 23.61 ppm;HRMS (EI, m/z): [M+H]+ calculated for C_24_H_19_BrN_4_O_3_490.0641, Found490.0641.

**5-Phenyl-3-((1-(4-(trifluoromethyl)phenyl)-1H-1,2,3-triazol-4-yl)methyl)-1-oxa-5-azaspiro[5.5]undeca-7,10-diene-4,9-dione(2f):**

Pale yellow liquid; 89% yield; purity 97.9%; IR (neat): 2920, 2860, 2698, 1718, 1678, 1568, 1438, 715 cm^-1^;^1^H NMR (600 MHz, CDCl_3_) δ 7.97 (s, 1H), 7.91 (d, *J* = 8.4 Hz, 2H), 7.83 (d, *J* = 8.5 Hz, 2H), 7.28-7.34 (m, 3H), 7.19 (dd, *J* = 10.3, 3.2 Hz, 1H), 7.01 – 6.97 (m, 2H), 6.84 (dd, *J* = 10.2, 3.2 Hz, 1H), 6.13 (dd, *J* = 10.3, 2.0 Hz, 1H), 6.04 (dd, *J* = 10.2, 2.0 Hz, 1H), 4.64 (dd, *J* = 12.2, 9.7 Hz, 1H), 4.42 (dd, *J* = 12.2, 6.5 Hz, 1H), 3.45 (dd, *J* = 15.0, 6.7 Hz, 1H), 3.40 – 3.33 (m, 1H), 3.21 (dd, *J* = 15.0, 3.9 Hz, 1H); ^13^C NMR (150 MHz, CDCl_3_) δ 183.93, 169.52, 145.69, 144.65, 142.77, 129.90, 129.86, 129.75 (2 C), 129.12 (4 C), 129.06, 127.16, 127.14, 124.42, 120.82, 120.77, 120.28 (2 C), 83.48, 63.73, 41.53, 23.61 ppm;HRMS (EI, m/z): [M+H]+ calculated for C_25_H_19_F_3_N_4_O_3_480.1409, Found 480.1408.

**3-(4-((4,9-Dioxo-5-phenyl-1-oxa-5-azaspiro[5.5]undeca-7,10-dien-3-yl)methyl)-1H-1,2,3-triazol-1-yl)benzonitrile(2g):**

Brown liquid; 87% yield; purity 97.8%; IR (neat): 2932, 2867, 2678, 1715, 1658, 1430, 715 cm^-1^;^1^H NMR (600 MHz, CDCl_3_) δ 8.09 – 8.05 (m, 1H), 8.03 – 7.97 (m, 1H), 7.93 (s, 1H), 7.75 – 7.70 (m, 1H), 7.66 (dd, *J* = 12.9, 4.9 Hz, 1H), 7.31-7.26 (m, 3H), 7.17 (dd, *J* = 10.3, 3.2 Hz, 1H), 6.99 – 6.95 (m, 2H), 6.82 (dd, *J* = 10.2, 3.2 Hz, 1H), 6.11 (dd, *J* = 10.3, 2.0 Hz, 1H), 6.02 (dd, *J* = 10.2, 2.0 Hz, 1H), 4.59 (dd, *J* = 12.1, 9.6 Hz, 1H), 4.39 (dd, *J* = 12.2, 6.4 Hz, 1H), 3.41 (dd, *J* = 15.0, 6.7 Hz, 1H), 3.38 – 3.32 (m, 1H), 3.18 (dd, *J* = 15.0, 4.1 Hz, 1H); ^13^C NMR (150 MHz, CDCl_3_) δ 183.72, 169.37, 144.39, 142.54, 136.12, 131.84, 130.71, 129.72, 129.69 (2 C), 129.54, 128.93 (4 C), 128.90, 128.79, 124.01, 123.30, 119.70, 114.06, 83.31, 63.53, 41.30, 23.43 ppm;HRMS (EI, m/z): [M+H]+ calculated for C_25_H_19_N_5_O_3_437.1488, Found 437.1478.

**3-((1-(4-Methoxyphenyl)-1H-1,2,3-triazol-4-yl)methyl)-5-phenyl-1-oxa-5-azaspiro[5.5]undeca-7,10-diene-4,9-dione(2h):**

Yellow liquid; 93% yield; purity 98.8%; IR (neat): 2920, 2851, 1728, 1678, 1518, 1392, 1094, 698 cm^-1^;^1^H NMR (600 MHz, CDCl_3_) δ 7.80 (s, 1H), 7.64 (d, *J* = 9.0 Hz, 2H), 7.32 – 7.28 (m, 3H), 7.19 (dd, *J* = 10.3, 3.2 Hz, 1H), 7.04 (d, *J* = 9.0 Hz, 2H), 6.98 (dd, *J* = 7.7, 1.5 Hz, 2H), 6.84 (dd, *J* = 10.2, 3.2 Hz, 1H), 6.12 (dd, *J* = 10.3, 2.0 Hz, 1H), 6.03 (dd, *J* = 10.2, 2.0 Hz, 1H), 4.67 (dd, *J* = 12.2, 9.7 Hz, 1H), 4.40 (dd, *J* = 12.2, 6.6 Hz, 1H), 3.90 (s, 3H), 3.44 (dd, *J* = 15.0, 6.9 Hz, 1H), 3.40 – 3.31 (m, 1H), 3.18 (dd, *J* = 15.0, 3.7 Hz, 1H); ^13^C NMR (150 MHz, CDCl_3_) δ 184.02, 169.59, 159.83, 144.78, 142.96, 136.47, 130.44, 129.83, 129.80 (3 C), 129.07 (2 C), 128.97 (2 C), 122.02 (2 C), 121.11, 114.80 (2 C), 83.43, 63.74, 55.65, 41.54, 31.94 ppm;HRMS (EI, m/z): [M+H]+ calculated for C_25_H_22_N_4_O_4_442.1641, Found 442.1637.

**3-((1-(4-(Methylthio)phenyl)-1H-1,2,3-triazol-4-yl)methyl)-5-phenyl-1-oxa-5-azaspiro[5.5]undeca-7,10-diene-4,9-dione(2i):**

Dark yellow liquid; 92% yield; purity 98.3%; IR (neat): 2996, 2838, 2748, 1718, 1680, 1520, 1379, 715 cm^-1^;^1^H NMR (600 MHz, CDCl_3_) δ 7.97 – 7.94 (m, 1H), 7.85 (s, 1H), 7.66 (d, *J* = 8.6 Hz, 2H), 7.39 (d, *J* = 8.7 Hz, 1H), 7.34 – 7.29 (m, 3H), 7.18 (dd, *J* = 10.3, 3.3 Hz, 1H), 6.98 (t, *J* = 6.1 Hz, 2H), 6.84 (dd, *J* = 10.2, 3.2 Hz, 1H), 6.12 (dd, *J* = 10.3, 2.0 Hz, 1H), 6.03 (dd, *J* = 10.2, 2.0 Hz, 1H), 4.65 (dd, *J* = 12.1, 9.6 Hz, 1H), 4.43 – 4.33 (m, 1H), 3.44 (ddd, *J* = 15.0, 6.7, 3.2 Hz, 1H), 3.39 – 3.32 (m, 1H), 3.18 (dd, *J* = 15.1, 3.7 Hz, 1H); 2.66 (s, 3H)^13^C NMR (150 MHz, CDCl_3_) δ 183.81, 169.39, 144.55, 142.71, 139.79, 136.24, 129.65, 129.62, 129.59, 129.57 (2 C), 128.92, 128.89 (4 C), 128.81, 126.97, 120.56, 83.25, 63.53, 41.34, 31.75, 22.51, 13.94 ppm;HRMS (EI, m/z): [M+H]+ calculated for C_25_H_22_N_4_O_3_S459.1485, Found 459.1481.

**3-((1-(3-Benzylphenyl)-1H-1,2,3-triazol-4-yl)methyl)-5-phenyl-1-oxa-5-azaspiro[5.5]undeca-7,10-diene-4,9-dione(2j):**

Pale brown liquid; 84% yield; purity 98.2%; IR (neat): 2960, 2920, 2836, 2789, 2672, 1715, 1587, 1314, 697 cm^-1^;^1^H NMR (600 MHz, CDCl_3_) δ 7.82 (s, 1H), 7.57 (s, 1H), 7.54 (d, *J* = 8.0 Hz, 1H), 7.43 (t, *J* = 7.8 Hz, 1H), 7.35 – 7.29 (m, 2H), 7.28 (d, *J* = 7.2 Hz, 2H), 7.25-7.19 (m, 5H), 7.15 (dd, *J* = 10.3, 3.2 Hz, 1H), 6.95 (d, *J* = 7.1 Hz, 2H), 6.81 (dd, *J* = 10.2, 3.2 Hz, 1H), 6.09 (dd, *J* = 10.3, 2.0 Hz, 1H), 6.01 (dd, *J* = 10.2, 2.0 Hz, 1H), 4.62 (dd, *J* = 12.2, 9.7 Hz, 1H), 4.37 (dd, *J* = 12.2, 6.6 Hz, 1H), 4.06 (s, 2H), 3.41 (dd, *J* = 15.0, 6.9 Hz, 1H), 3.35 – 3.27 (m, 1H), 3.15 (dd, *J* = 14.9, 3.7 Hz, 1H); ^13^C NMR (150 MHz, CDCl_3_) δ 184.01, 169.58, 144.95, 144.75, 143.37, 142.93, 139.96, 137.12, 136.43, 129.85, 129.83, 129.80, 129.79 (2 C), 129.40, 129.07 (2 C), 128.98, 128.91 (2 C), 128.71 (2 C), 126.54, 121.05, 120.92, 118.21, 83.43, 63.70, 41.75, 41.54, 23.63;HRMS (EI, m/z): [M+H]+ calculated for C_31_H_26_N_4_O_3_502.2005, Found 502.2008.

**5-Phenyl-3-((1-(4-((trifluoromethyl)sulfonyl)phenyl)-1H-1,2,3-triazol-4-yl)methyl)-1-oxa-5-azaspiro[5.5]undeca-7,10-diene-4,9-dione (2k):**

Light Yellow liquid; 80% yield; purity 97.8%; IR (neat): 2960, 2951, 2852, 2790, 2672, 1720, 1687, 1214, 790 cm^-11^H NMR (600 MHz, CDCl_3_) δ 8.22 (d, *J* = 8.7 Hz, 2H), 8.10 (d, *J* = 8.8 Hz, 2H), 8.05 (s, 1H), 7.33 – 7.27 (m, 4H), 7.16 (dd, *J* = 10.3, 3.2 Hz, 1H), 6.98 (d, *J* = 6.6 Hz, 2H), 6.83 (dd, *J* = 10.2, 3.2 Hz, 1H), 6.12 (dd, *J* = 10.3, 2.0 Hz, 1H), 6.03 (dd, *J* = 10.2, 2.0 Hz, 1H), 4.58 (dd, *J* = 12.1, 9.6 Hz, 1H), 4.40 (dd, *J* = 12.1, 6.4 Hz, 1H), 3.42 (dd, *J* = 15.0, 6.6 Hz, 1H), 3.38 – 3.33 (m, 1H), 3.21 (dd, *J* = 15.0, 4.2 Hz, 1H); ^13^C NMR (150 MHz, CDCl_3_) δ 183.95, 169.59, 146.49, 144.59, 142.73, 142.70, 136.35, 133.00, 130.90, 130.03, 129.99, 129.80 (3 C), 129.22 (4 C), 129.08, 120.77,118.65-122.77 (m, CF3),83.60, 63.80, 41.58, 23.70 ppm; HRMS (EI, m/z): [M+H]+ calculated for C_25_H_19_F_3_N_4_O_5_S544.1028, Found 544.1011.

**N-(4-(4-((4,9-Dioxo-5-phenyl-1-oxa-5-azaspiro[5.5]undeca-7,10-dien-3-yl)methyl)-1H-1,2,3-triazol-1-yl)phenyl)acetamide(2l):**

Brown gummy liquid; 78% yield; purity 97.7%; IR (neat): 3246, 2989, 2942, 2780, 2670, 1720, 1688, 1314, 710 cm^-1^; ^1^H NMR (600 MHz, CDCl_3_) δ 7.82 (s, 1H), 7.69-7.63 (m, 3H), 7.47 (s, 1H), 7.33 – 7.28 (m, 4H), 7.24 (dd, *J* = 10.3, 3.1 Hz, 1H), 7.00 (d, *J* = 6.3 Hz, 2H), 6.84 (dd, *J* = 10.2, 3.2 Hz, 1H), 6.14 (dd, *J* = 10.3, 2.0 Hz, 1H), 6.04 (dd, *J* = 10.2, 2.0 Hz, 1H), 4.68 (dd, *J* = 12.1, 9.8 Hz, 1H), 4.40 (dd, *J* = 12.2, 6.6 Hz, 1H), 3.45 (dd, *J* = 15.1, 6.8 Hz, 1H), 3.39-3.33 (m, 1H), 3.17 (dd, *J* = 15.1, 3.7 Hz, 1H), 2.22 (s, 3H); ^13^C NMR (150 MHz, CDCl_3_) δ 181.77, 169.46, 168.20, 144.75, 144.53 (2 C), 142.71, 136.22, 129.70, 129.66 (2 C), 129.63, 129.58, 128.88 (4 C), 128.82, 120.84, 120.60, 120.30, 83.26, 63.52, 41.21, 23.36, 22.50 ppm;HRMS (EI, m/z): [M+H]+ calculated for C_25_H_19_F_3_N_4_O_5_S469.1750, Found469.1746.

**3-((1-(3,5-B=Dimethoxyphenyl)-1H-1,2,3-triazol-4-yl)methyl)-5-phenyl-1-oxa-5-azaspiro[5.5]undeca-7,10-diene-4,9-dione(2m):**

Pale yellow liquid; 74% yield; purity 97.8%; IR (neat): 2980, 2842, 2765, 2679, 1708, 1687, 1414, 710 cm^-1^; ^1^H NMR (600 MHz, CDCl_3_) δ 7.83 (s, 1H), 7.33 – 7.27 (m, 4H), 7.15 (dd, *J* = 10.3, 3.2 Hz, 1H), 6.97 (dd, *J* = 8.8, 2.8 Hz, 2H), 6.88 (d, *J* = 2.2 Hz, 1H), 6.82 (dd, *J* = 10.2, 3.2 Hz, 1H), 6.52 (t, *J* = 2.2 Hz, 1H), 6.11 (dd, *J* = 10.3, 2.0 Hz, 1H), 6.02 (dd, *J* = 10.2, 2.0 Hz, 1H), 4.63 (dd, *J* = 12.2, 9.7 Hz, 1H), 4.38 (dd, *J* = 12.2, 6.6 Hz, 1H), 3.86 (s, 6H), 3.46 – 3.39 (m, 1H), 3.34 (ddd, *J* = 12.8, 6.6, 3.3 Hz, 1H), 3.17 (dd, *J* = 15.0, 3.8 Hz, 1H); ^13^C NMR (151 MHz, CDCl_3_) δ 182.95, 168.54, 147.10, 147.03, 143.69, 141.85, 135.39, 128.82 (2 C), 128.79 (2 C), 128.75 (2 C), 128.06, 127.98, 119.96, 115.09 (2 C), 112.69, 108.43, 82.42, 62.70, 55.51 (2 C), 40.49, 22.58 ppm;HRMS (EI, m/z): [M+H]+ calculated for C_26_H_24_N_4_O_5_472.1747, Found 472.1749.

**3-((1-(4-fluoro-3-methoxyphenyl)-1H-1,2,3-triazol-4-yl)methyl)-5-phenyl-1-oxa-5-azaspiro[5.5]undeca-7,10-diene-4,9-dione(2n):**

Dark yellow liquid; 74% yield; purity 98.2%; IR (neat): 2984, 2832, 2865, 2568, 1718, 1584, 1320, 697 cm^-1^; ^1^H NMR (600 MHz, CDCl_3_) δ 7.81 (s, 1H), 7.55 (dd, *J* = 11.3, 2.5 Hz, 1H), 7.46 – 7.41 (m, 1H), 7.31 (dd, *J* = 11.9, 6.9 Hz, 3H), 7.19 (dd, *J* = 10.3, 3.2 Hz, 1H), 7.09 (t, *J* = 8.7 Hz, 1H), 7.01 – 6.97 (m, 2H), 6.84 (dd, *J* = 10.2, 3.2 Hz, 1H), 6.13 (dd, *J* = 10.3, 1.9 Hz, 1H), 6.04 (dd, *J* = 10.2, 2.0 Hz, 1H), 4.64 (dd, *J* = 12.1, 9.7 Hz, 1H), 4.40 (dd, *J* = 12.2, 6.6 Hz, 1H), 3.98 (s, 3H), 3.43 (dd, *J* = 14.9, 6.8 Hz, 1H), 3.38-3.32 (m, 1H), 3.18 (dd, *J* = 14.9, 3.7 Hz, 1H); ^13^C NMR (150 MHz, CDCl_3_) δ 183.93, 169.52, 148.04 (d, *J* = 10.7 Hz, C), 144.67, 142.83, 136.37, 129.80, 129.77, 129.73 (2 C), 129.04 (4 C), 128.96, 120.94, 116.06, 113.67, 109.41, 83.40, 63.68, 56.49, 41.47, 22.65 ppm;HRMS (EI, m/z): [M+H]+ calculated for C_25_H_21_FN_4_O_4_460.1547, Found 460.1548.

**3-((1-(benzo[d][1,3]dioxol-5-yl)-1H-1,2,3-triazol-4-yl)methyl)-5-phenyl-1-oxa-5-azaspiro[5.5]undeca-7,10-diene-4,9-dione(2o):**

Pale dark liquid; 96% yield; purity 97.6%; IR (neat): 2918, 2852, 2825, 1715, 1679, 1584, 1320, 697 cm^-1^; ^1^H NMR (600 MHz, CDCl_3_) δ 7.78 (s, 1H), 7.34 – 7.29 (m, 3H), 7.26 (d, *J* = 2.2 Hz, 1H), 7.19 (dd, *J* = 10.3, 3.2 Hz, 1H), 7.13 (dd, *J* = 8.3, 2.2 Hz, 1H), 7.01 – 6.96 (m, 2H), 6.92 (d, *J* = 8.3 Hz, 1H), 6.84 (dd, *J* = 10.2, 3.2 Hz, 1H), 6.13 (dd, *J* = 10.3, 2.0 Hz, 1H), 6.10 (s, 2H), 6.04 (dd, *J* = 10.2, 2.0 Hz, 1H), 4.65 (dd, *J* = 12.2, 9.6 Hz, 1H), 4.40 (dd, *J* = 12.2, 6.6 Hz, 1H), 3.43 (dd, *J* = 15.0, 6.9 Hz, 1H), 3.38 – 3.32 (m, 1H), 3.17 (dd, *J* = 14.9, 3.7 Hz, 1H); ^13^C NMR (150 MHz, CDCl_3_) δ 180.08, 169.59, 144.84, 144.75, 144.28, 142.92, 136.43, 129.83, 129.80, 129.79 (2 C), 129.08 (4 C), 128.99, 121.24, 114.04, 108.52, 102.59, 102.13, 83.43, 63.72, 41.51, 22.70 ppm; HRMS (EI, m/z): [M+H]+ calculated for C_25_H_20_N_4_O_5_456.1434, Found 456.1431.

**3-((1-(4-methyl-2-oxo-2H-chromen-7-yl)-1H-1,2,3-triazol-4-yl)methyl)-5-phenyl-1-oxa-5-azaspiro[5.5]undeca-7,10-diene-4,9-dione(2p):**

Brown gummy liquid; 85% yield; purity 98.8%; IR (neat): 2988, 2862, 2718, 1740, 1710, 1689, 1489, 1270, 1098, 733 cm^-1^;^1^H NMR (600 MHz, CDCl_3_) δ 7.96 (s, 1H), 7.78 – 7.73 (m, 2H), 7.71 (d, *J* = 1.1 Hz, 1H), 7.33 – 7.27 (m, 3H), 7.19 (dd, *J* = 10.3, 3.2 Hz, 1H), 7.02 – 6.96 (m, 2H), 6.83 (dd, *J* = 10.2, 3.2 Hz, 1H), 6.37 (d, *J* = 1.1 Hz, 1H), 6.12 (dd, *J* = 10.3, 2.0 Hz, 1H), 6.03 (dd, *J* = 10.2, 2.0 Hz, 1H), 4.62 (dd, *J* = 12.1, 9.5 Hz, 1H), 4.40 (dd, *J* = 12.2, 6.4 Hz, 1H), 3.43 (dd, *J* = 15.0, 6.8 Hz, 1H), 3.38-3.32 (m, 1H), 3.20 (dd, *J* = 15.0, 3.9 Hz, 1H), 2.49 (d, *J* = 1.1 Hz, 3H); ^13^C NMR (150 MHz, CDCl_3_) δ 184.00, 169.59, 159.96, 151.51, 144.68, 142.89, 136.41, 133.40, 129.94, 129.90, 129.81 (3 C), 129.17 (4 C), 129.11, 126.29, 120.81, 115.78, 115.74, 108.40, 83.53, 63.80, 41.51, 23.67, 18.76 ppm;HRMS (EI, m/z): [M+H]+ calculated for C_28_H_22_N_4_O_5_494.1590, Found494.1575.

**3-((1-((3R,5S)-adamantan-1-yl)-1H-1,2,3-triazol-4-yl)methyl)-5-phenyl-1-oxa-5-azaspiro[5.5]undeca-7,10-diene-4,9-dione(2q)**

Light brown liquid; 89% yield; purity 98.3%; IR (neat): 2957, 2922, 2852, 1708, 1690, 1592, 1486, 1356, 760, 697 cm^-1^;^1^H NMR (600 MHz, CDCl_3_) δ 7.47 (s, 1H), 7.30 (d, *J* = 6.0 Hz, 3H), 7.09 (dd, *J* = 10.3, 3.1 Hz, 1H), 6.99 – 6.93 (m, 2H), 6.81 (dd, *J* = 10.2, 3.1 Hz, 1H), 6.11 (dd, *J* = 10.3, 1.8 Hz, 1H), 6.01 (dd, *J* = 10.2, 1.9 Hz, 1H), 4.65 (dd, *J* = 12.1, 9.8 Hz, 1H), 4.35 (dd, *J* = 12.2, 6.8 Hz, 1H), 3.37 (dd, *J* = 14.8, 6.9 Hz, 1H), 3.32-3.25 (m, 1H), 3.08 (dd, *J* = 14.8, 3.3 Hz, 1H), 2.29 (s, 3H), 2.25 (s, 6H), 1.82 (q, *J* = 12.5 Hz, 6H); ^13^C NMR (150 MHz, CDCl_3_) δ 184.08, 169.66, 144.86, 143.08, 136.57, 129.78 (2 C), 129.74, 129.70, 129.01 (2 C), 128.92, 123.98, 119.19, 83.32, 63.71, 59.50, 43.06 (2 C), 41.50, 35.91 (2 C), 31.94, 29.67, 29.44 (2 C), 29.37, 22.70 ppm; HRMS (EI, m/z): [M+H]+ calculated for C_28_H_30_N_4_O_3_470.2318, Found 470.2314.

**3-((1-Benzyl-1H-1,2,3-triazol-4-yl)methyl)-5-phenyl-1-oxa-5-azaspiro[5.5]undeca-7,10-diene-4,9-dione (2r):**

Brown liquid; 82% yield; purity 98.1%; IR (neat): 2989, 2939, 2852, 1715, 1590, 1448, 1378, 1170, 715, cm^-1^; ^1^H NMR (600 MHz, CDCl_3_) δ 7.43 – 7.37 (m, 4H), 7.34 – 7.29 (m, 3H), 7.25 (t, *J* = 7.4 Hz, 2H), 6.92 (dd, *J* = 10.3, 3.2 Hz, 1H), 6.83 (d, *J* = 7.3 Hz, 2H), 6.78 (dd, *J* = 10.2, 3.2 Hz, 1H), 6.06 (dd, *J* = 10.3, 2.0 Hz, 1H), 5.99 (dd, *J* = 10.2, 2.0 Hz, 1H), 5.59 (d, *J* = 14.8 Hz, 1H), 5.46 (d, *J* = 14.8 Hz, 1H), 4.49 (dd, *J* = 12.1, 9.8 Hz, 1H), 4.33 (dd, *J* = 12.2, 6.7 Hz, 1H), 3.38 (dd, *J* = 15.0, 6.5 Hz, 1H), 3.33 – 3.23 (m, 1H), 3.06 (dd, *J* = 15.0, 3.7 Hz, 1H); ^13^C NMR (151 MHz, CDCl_3_) δ 183.99, 177.33, 169.45, 144.71, 142.85, 136.39, 134.79, 130.50, 129.73, 129.67 (3 C), 129.18 (2 C), 129.04 (2 C), 128.90, 128.86, 128.06 (2 C), 83.27, 63.60, 54.20, 41.29, 23.61 ppm;HRMS (EI, m/z): [M+H]+ calculated for C_25_H_22_N_4_O_3_426.1692, Found 426.1687.

**Supplementary S2:** Redocking experiment to validate the performance of Docking program

**
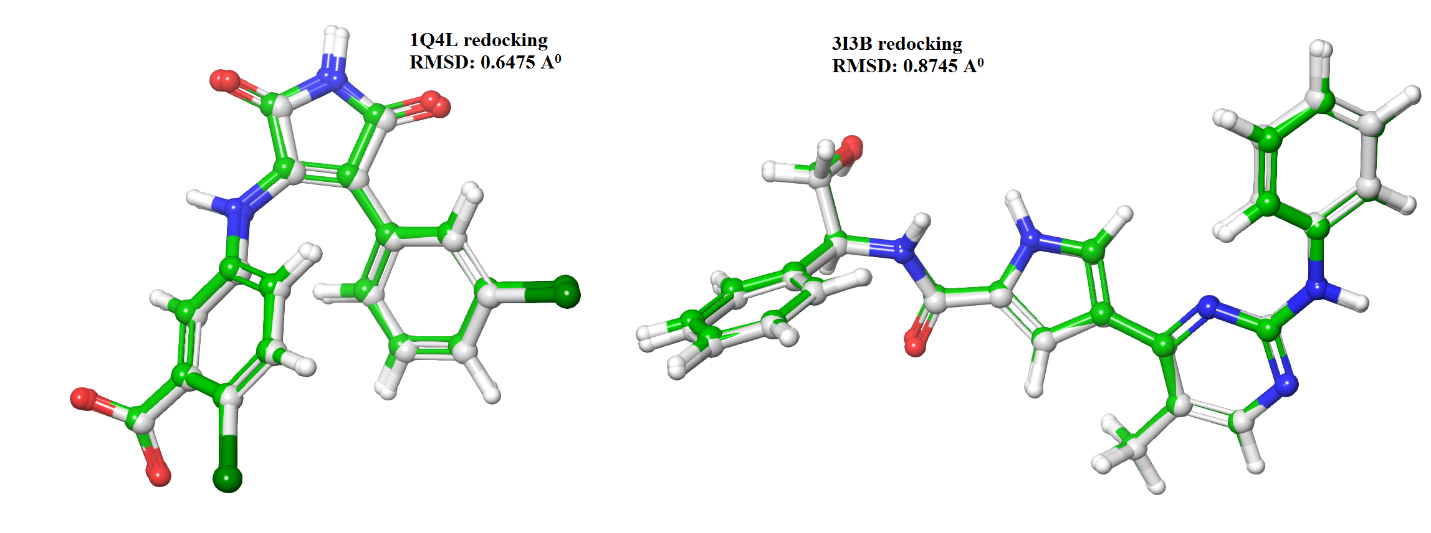
**

**Supplementary S3:** selected active inhibitors interaction with GSK3beta residue during MD simulation

| **Compound ID** | **Docking Score** | **Amino Acid Residues within 3 Å** | **Amino Acid Residues involved in direct interaction** | **Amino Acid Residues involved in Water Bridges** |
| --- | --- | --- | --- | --- |
| **2d** | -4.694 | Gly63,Ser66, Gly68, Val70, Lys85, Val87, Phe93, Leu132, Tyr134, Leu188, Cys199, ASP200 | Lys85 (π-Cation), Phe93(π-π), Tyr134 (H-Bond and π-π), | Gln185, Asp200, Val135 |
| **2g** | -4.841 | Gly63, Asn64, Ser66, Phe67, Gly68, Val70, Lys85, Val87, Leu88, Phe93, Leu132, Tyr134, Leu188, Cys199, ASP200 | Lys85 (π-Cation), Phe93(π-π), Tyr134, Leu88 and ASN64 (H-Bond and π-π), | Arg96, Glu97, Asp200, |
| **2j** | -5.743 | Gly63, Asn64, Ser66, Phe67, Gly68, Val70, Lys85, Val87, Leu88, Phe93, Leu132, Tyr134, Leu188, Cys199, ASP200 | Lys85 (π-Cation), Phe93 and Phe67 (π-π), Tyr134, Cyc199 and ASN64 (H-Bond and π-π), | Gln185, Ile62, Asp200, |
| **2k** | -4.918 | Ile62, Gly63, Asn64, Ser66, Phe67, Gly68, Val70, Ala83, Lys85, Arg92, Phe93, Lys94, Arg96, Leu132, Tyr134, Tyr140, Leu188, Cys199, ASP200 | Lys85 and Arg96 (π-Cation), Phe93 and Phe67 (π-π), Tyr134, Cyc199 and Lys94 (H-Bond and π-π), | Gln185, Arg92, Glu97, Ile62, Pro136, Asp200, |
| **2p** | -6.636 | Ile62, Asn64, Gly65, Ser66, Phe67, Gly68, Val70, Lys85, Val87,Leu88, Gln89, Asp90, Phe93, Leu132, Asp133, Tyr134, Val135, Tyr140, Leu188, Cys199, ASP200 | Lys85(π-Cation), Phe93 and Phe67 (π-π), Tyr134, Tyr140, Cyc199 and Leu88 (H-Bond and π-π), | Leu88, Asp133, Val135, Gln185, Asp200, |

**SupplementaryS4:**Figure representing the significance of contribution and interaction pattern at triazole substitution for active vs inactive


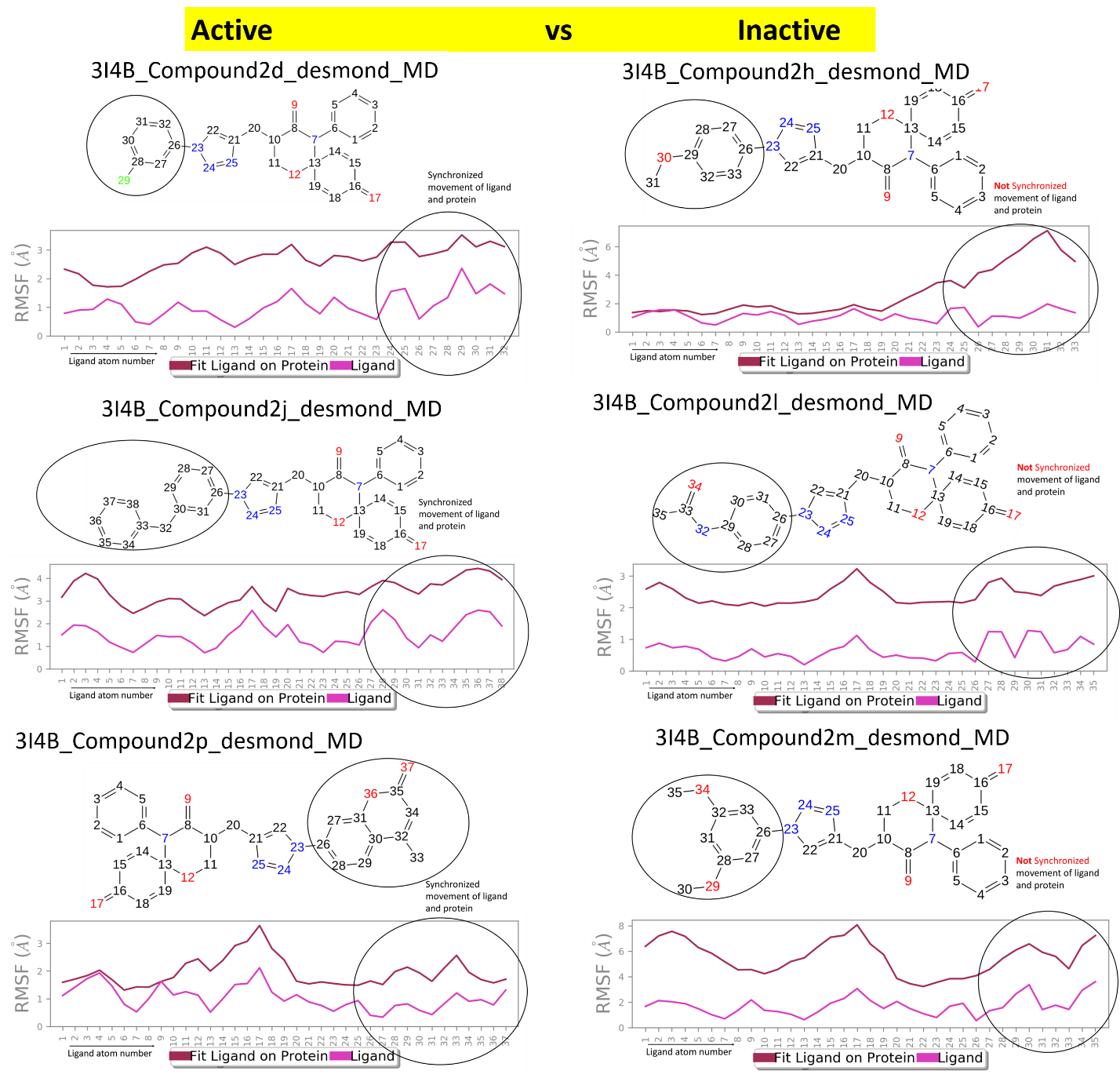


**Supplementary S5:** Representation of the RMSD of protein and respective ligand during the molecular dynamics simulation


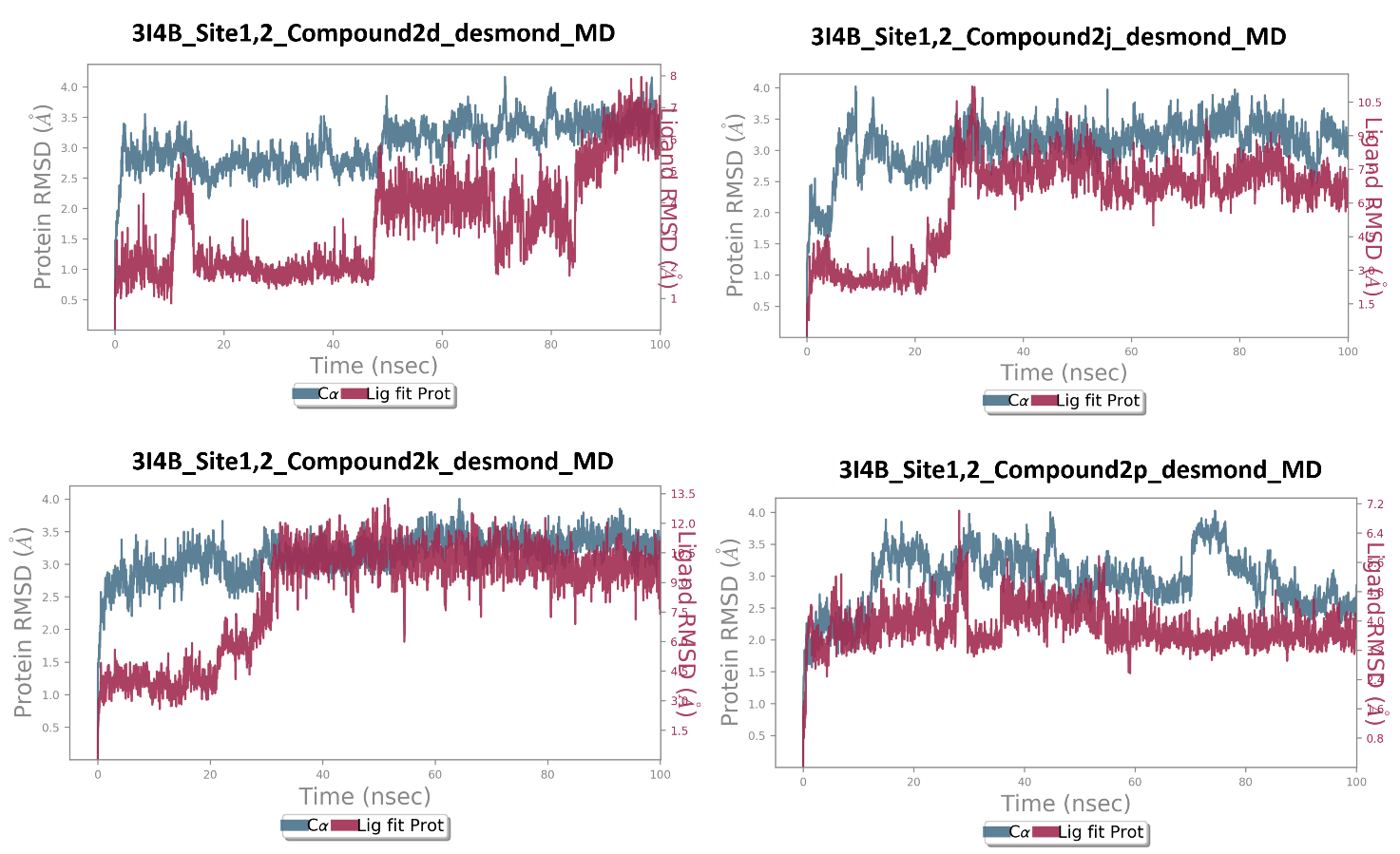


**Supplementary S6:** ADMET parameter of the synthesized compounds

| **No** | **glob** | **QPpolrz** | **QPlogPC16** | **QPlogPoct** | **QPlogPw** | **QPlogPo/w** | **QPlogS** | **CIQPlogS** | **QPlogHERG** | **QPPCaco** | **QPlogBB** | **QPPMDCK** | **QPlogKp** | **PercentHumanOralAbsorption** |
| --- | --- | --- | --- | --- | --- | --- | --- | --- | --- | --- | --- | --- | --- | --- |
| 1a | 0.835402 | 41.937 | 12.763 | 17.501 | 9.802 | 4.107 | -5.171 | -6.601 | -6.123 | 1608.916 | -0.162 | 2194.796 | -1.438 | 100 |
| 1b | 0.813044 | 53.749 | 16.482 | 24.488 | 14.756 | 3.725 | -4.69 | -6.704 | -7.243 | 591.207 | -1.04 | 280.403 | -1.435 | 100 |
| 7 | 0.867516 | 32.892 | 9.866 | 14.629 | 9.698 | 2.559 | -3.519 | -3.697 | -5.203 | 1610.731 | -0.294 | 828.166 | -1.732 | 100 |
| 2a | 0.813731 | 47.53 | 14.271 | 20.777 | 12.788 | 3.39 | -4.951 | -5.721 | -6.875 | 572.321 | -0.872 | 270.637 | -2.013 | 96.149 |
| 2b | 0.849522 | 45.412 | 13.553 | 20.978 | 13.791 | 2.183 | -3.384 | -5.046 | -5.922 | 344.119 | -0.979 | 156.166 | -2.719 | 85.128 |
| 2c | 0.81079 | 47.77 | 13.835 | 20.807 | 12.558 | 3.632 | -5.308 | -6.103 | -6.743 | 589.783 | -0.752 | 505.981 | -2.121 | 100 |
| 2d | 0.832756 | 47.664 | 14.322 | 21.091 | 12.164 | 3.751 | -5.128 | -6.453 | -6.147 | 634.38 | -0.592 | 745.65 | -2.237 | 100 |
| 2e | 0.803438 | 49.147 | 14.973 | 21.442 | 12.546 | 3.977 | -5.819 | -7.397 | -6.813 | 590.542 | -0.701 | 743.261 | -2.156 | 100 |
| 2f | 0.814536 | 49.807 | 13.483 | 21.882 | 12.185 | 4.383 | -5.978 | -7.165 | -6.463 | 898.33 | -0.374 | 1933.315 | -1.928 | 100 |
| 2g | 0.798286 | 49.148 | 15.162 | 22.168 | 14.385 | 2.576 | -5.704 | -6.557 | -6.982 | 132.561 | -1.684 | 55.691 | -3.258 | 80.013 |
| 2h | 0.803589 | 49.352 | 14.573 | 22.083 | 13.002 | 3.493 | -5.075 | -6.011 | -6.798 | 664.608 | -0.895 | 318.101 | -1.969 | 100 |
| 2i | 0.792619 | 50.992 | 15.24 | 22.386 | 12.76 | 4.046 | -5.885 | -6.468 | -6.923 | 660.905 | -0.829 | 539.628 | -2.013 | 100 |
| 2j | 0.774764 | 58.776 | 17.823 | 24.898 | 13.391 | 5.41 | -7.315 | -7.995 | -8.011 | 606.663 | -1.098 | 288.232 | -1.399 | 82.514 |
| 2k | 0.801225 | 53.431 | 15.012 | 25.54 | 16.437 | 2.591 | -4.657 | -6.67 | -6.616 | 180.138 | -1.258 | 302.911 | -3.234 | 69.53 |
| 2l | 0.802409 | 51.77 | 15.55 | 25.416 | 16.337 | 2.938 | -5.628 | -6.103 | -6.587 | 178.001 | -1.523 | 76.585 | -3.252 | 84.427 |
| 2m | 0.789681 | 51.195 | 14.958 | 22.709 | 13.264 | 3.527 | -5.288 | -6.301 | -6.756 | 608.775 | -1.043 | 289.316 | -2.145 | 100 |
| 2n | 0.800835 | 49.669 | 14.203 | 21.786 | 12.799 | 3.721 | -5.427 | -6.393 | -6.672 | 655.283 | -0.803 | 544.575 | -2.107 | 100 |
| 2o | 0.85532 | 47.125 | 13.59 | 21.325 | 13.424 | 2.668 | -3.633 | -6.075 | -5.683 | 635.122 | -0.704 | 302.874 | -2.31 | 92.734 |
| 2p | 0.786802 | 54.507 | 16.189 | 24.703 | 15.568 | 2.874 | -5.403 | -6.544 | -7.063 | 135.952 | -1.672 | 57.232 | -3.41 | 81.956 |
| 2q | 0.851455 | 50.392 | 13.559 | 21.466 | 11.057 | 4.052 | -5.22 | -6.547 | -5.097 | 787.903 | -0.623 | 382.34 | -2.606 | 100 |
| 2r | 0.871186 | 45.022 | 13.445 | 20.009 | 11.996 | 3.056 | -3.45 | -5.787 | -5.485 | 717.579 | -0.734 | 345.591 | -1.971 | 95.951 |

^Predictions for Properties:^

^QP Polarizability (Angstroms^3) = 53.749M ( 13.0 / 70.0)^

^QP log P for hexadecane/gas = 16.482M ( 4.0 / 18.0)^

^QP log P for octanol/gas = 24.488M ( 8.0 / 35.0)^

^QP log P for water/gas = 14.756M ( 4.0 / 45.0)^

^QP log P for octanol/water = 3.725 ( -2.0 / 6.5)^

^QP log S for aqueous solubility = -4.690 ( -6.5 / 0.5)^

^QP log S - conformation independent = -6.704 ( -6.5 / 0.5)^

^QP log K hsa Serum Protein Binding = -0.014 ( -1.5 / 1.5)^

^QP log BB for brain/blood = -1.040 ( -3.0 / 1.2)^

^HERG K+ Channel Blockage: log IC50 = -7.243 (concern below -5)^

^Apparent Caco-2 Permeability (nm/sec) = 591 (<25 poor, >500 great)^

^Apparent MDCK Permeability (nm/sec) = 280M (<25 poor, >500 great)^

^QP log Kp for skin permeability = -1.435 (Kp in cm/hr)^

^% Human Oral Absorption in GI (+-20%) = 100 (<25% is poor)^

^Qual. Model for Human Oral Absorption = HIGH (>80% is high)^

**Supplementary S7:** Kinase panel screening of compound **2d**

|  | % Enzyme Activity (relative to DMSO controls) | | IC50 (M) of Control (+) | Control(+) |
| --- | --- | --- | --- | --- |
|  | **Compound 2d** | |  |  |
| Kinase: | **Data 1** | **Data 2** |  |  |
| AMPK(A1/B1/G1) | 60.89 | 60.04 | 5.77E-12 | Staurosporine |
| AMPK(A1/B1/G2) | 68.01 | 66.48 | 3.68E-11 | Staurosporine |
| AMPK(A1/B1/G3) | 101.13 | 100.85 | 3.62E-10 | Staurosporine |
| AMPK(A1/B2/G1) | 109.51 | 107.43 | 3.21E-11 | Staurosporine |
| AMPK(A2/B1/G1) | **50.81** | **47.50** | 2.65E-11 | Staurosporine |
| AMPK(A2/B2/G1) | 94.05 | 89.86 | 4.59E-11 | Staurosporine |
| AMPK(A2/B2/G2) | 113.71 | 111.36 | 5.47E-11 | Staurosporine |
| AMPK(A2/B2/G3) | 95.82 | 88.96 | 1.36E-10 | Staurosporine |
| ARK5/NUAK1 | 93.57 | 84.16 | 4.75E-10 | Staurosporine |
| CDK1/cyclin B | **59.28** | **58.64** | 1.36E-09 | Staurosporine |
| CDK2/cyclin O | **54.36** | **54.15** | 1.57E-09 | Staurosporine |
| CDK3/cyclin E | **61.64** | **59.02** | 2.00E-09 | Staurosporine |
| DAPK1 | 75.82 | 73.41 | 2.88E-09 | Staurosporine |
| DAPK2 | 110.63 | 109.81 | 1.03E-08 | Staurosporine |
| DNA-PK | 102.84 | 101.60 | 1.57E-08 | PI-103 |
| EEF2K | 103.09 | 101.96 | 2.41E-05 | NH125 |
| EIF2AK1 | 91.72 | 87.95 | 1.06E-07 | GSK-2606414 |
| EIF2AK2 | 101.35 | 100.73 | 2.28E-08 | Staurosporine |
| EIF2AK3 | 70.78 | 70.64 | 5.40E-10 | GSK-2606414 |
| EIF2AK4 | 103.68 | 102.49 | 6.98E-07 | Staurosporine |
| GSK3b | **2.81** | **1.64** | 3.34E-09 | Staurosporine |
| Haspin | 71.58 | 70.31 | 1.65E-08 | Staurosporine |
| JNK1 | 82.70 | 82.67 | 1.99E-07 | Staurosporine |
| JNK2 | 80.30 | 79.67 | 3.62E-07 | Staurosporine |
| JNK3 | 95.45 | 95.42 | 1.28E-08 | JNKi VIII |
| MST3/STK24 | 94.20 | 89.43 | 1.03E-08 | Staurosporine |
| mTOR/FRAP1 | 103.16 | 96.09 | 7.48E-08 | PI-103 |
| P38b/MAPK11 | 110.21 | 109.52 | 3.11E-08 | SB202190 |
| PDK1/PDHK1 | 85.62 | 85.01 | 1.21E-05 | GW5074 |
| PDK2/PDHK2 | **64.28** | **63.30** | 3.28E-06 | GW5074 |
| PDK3/PDHK3 | 85.93 | 82.28 | 1.82E-06 | GW5074 |
| PDK4/PDHK4 | 111.00 | 105.05 | 2.12E-06 | GW5074 |
| PKCa | **62.98** | **62.24** | 3.33E-10 | Staurosporine |
| PKN2/PRK2 | 101.83 | 97.21 | 1.61E-09 | Staurosporine |
| RIPK5 | 86.12 | 83.85 | 2.29E-08 | Staurosporine |
| ROCK1 | 105.45 | 101.05 | 4.22E-10 | Staurosporine |
| ROCK2 | 100.46 | 98.38 | 2.87E-10 | Staurosporine |
| STK25/YSK1 | **63.29** | **61.07** | 2.63E-09 | Staurosporine |
| TLK1 | 82.06 | 81.34 | 1.01E-08 | Staurosporine |
| TRKA | 119.15 | 115.47 | 3.68E-09 | Staurosporine |
| TRKA (G595R) | 102.66 | 99.08 | 1.02E-09 | Staurosporine |
| TRKA (G667C) | 103.46 | 101.95 | 3.99E-10 | Staurosporine |
| TRKA-TFG (TRK-T3) | 109.31 | 102.82 | 1.06E-10 | Staurosporine |
| TRKA-TPM3 | 98.17 | 95.56 | 6.87E-11 | Staurosporine |
| TRKA-TPR | 105.68 | 101.99 | 9.57E-10 | Staurosporine |
| TRKB | 103.12 | 101.29 | 4.86E-11 | Staurosporine |
| TRKC | 108.18 | 102.60 | 8.61E-11 | Staurosporine |
| TRKC (G623E) | 117.78 | 114.78 | 3.22E-08 | Staurosporine |
| TRKC (G623R) | 92.10 | 90.32 | 9.81E-09 | Staurosporine |
| TRKC (G623R/L686M) | 96.00 | 95.31 | 5.81E-08 | Staurosporine |
| TRKC (L686M) | 107.04 | 106.77 | 1.22E-08 | Staurosporine |
| TRPM7/CHAK1 | **67.49** | **63.76** | 5.86E-06 | NH125 |
| ZIPK/DAPK3 | 88.84 | 86.95 | 4.43E-09 | Staurosporine |
| PI3KC2A | 121.53 | 123.51 | 4.80E-07 | PI-103 |
| PI3KC3 | 99.80 | 98.59 | 7.57E-08 | PIK-93 |
| PI4Ka | 100.96 | 101.50 | 4.50E-07 | PIK-93 |
| PI4Kb | 81.56 | 81.00 | 5.87E-09 | PIK-93 |
| PI4K2A | 75.88 | 74.47 | 1.85E-05 | PIK-93 |
| PIP5K1A | 86.01 | 84.84 | 6.18E-05 | PIK-93 |
| PIP5K1C | 88.16 | 87.90 | 1.75E-05 | PIK-93 |
| SPHK1 | 80.49 | 77.68 | 1.72E-08 | PF-543 |
| SPHK2 | 91.48 | 90.95 | 6.30E-05 | PF-543 |
| PI3Ka (p110a/p85a) | 252.16 | 250.92 | 4.61E-09 | PI-103 |
| PI3Kb (p110b/p85a) | 86.27 | 86.58 | 9.39E-09 | PI-103 |
| PI3Kg (p110g) | 87.40 | 85.15 | 4.89E-08 | PI-103 |
| PI3Kd (p110d/p85a) | 108.90 | 108.46 | 3.67E-09 | PI-103 |
| PI3K (p110a/p65a) | 83.76 | 82.25 | 3.67E-09 | PI-103 |
| PI3K (p110a(E542K)/p85a) | 102.87 | 100.20 | 4.39E-09 | PI-103 |
| PI3K (p110a(E545K)/p85a) | 206.13 | 206.92 | 3.54E-09 | PI-103 |
| PI3K (p110a(H1047R)/p85a) | 132.36 | 131.08 | 5.97E-09 | PI-103 |

**Supplementary S8:**NO production assay & cell viability of chosen compounds

| **No** | **Sample codes** | **^a^Cell Viability (% of LPS)** |
| --- | --- | --- |
| **1** | **1a** | **95.03±6.87** |
| **2** | **2a** | **92.90±0.24** |
| **5** | **2d** | **112.33±19.75** |
| **6** | **2e** | **106.03±1.28** |
| **11** | **2j** | **107.38±6.88** |
| **19** | **2r** | **101.27±0.67** |
| **^b^L-NMMA** | **Positive control** | **100.55±1.60** |

^a^cell viability after treatment with 1 μM of each compound was determined by MTT assay and is expressed in percentage (% of LPS). The results are averages of three independent experiments, and the data are expressed as mean ± SD; ^b^L-NMMA as positive control.

**Supplementary S9:** Original blots of compounds **2a** and **2d**


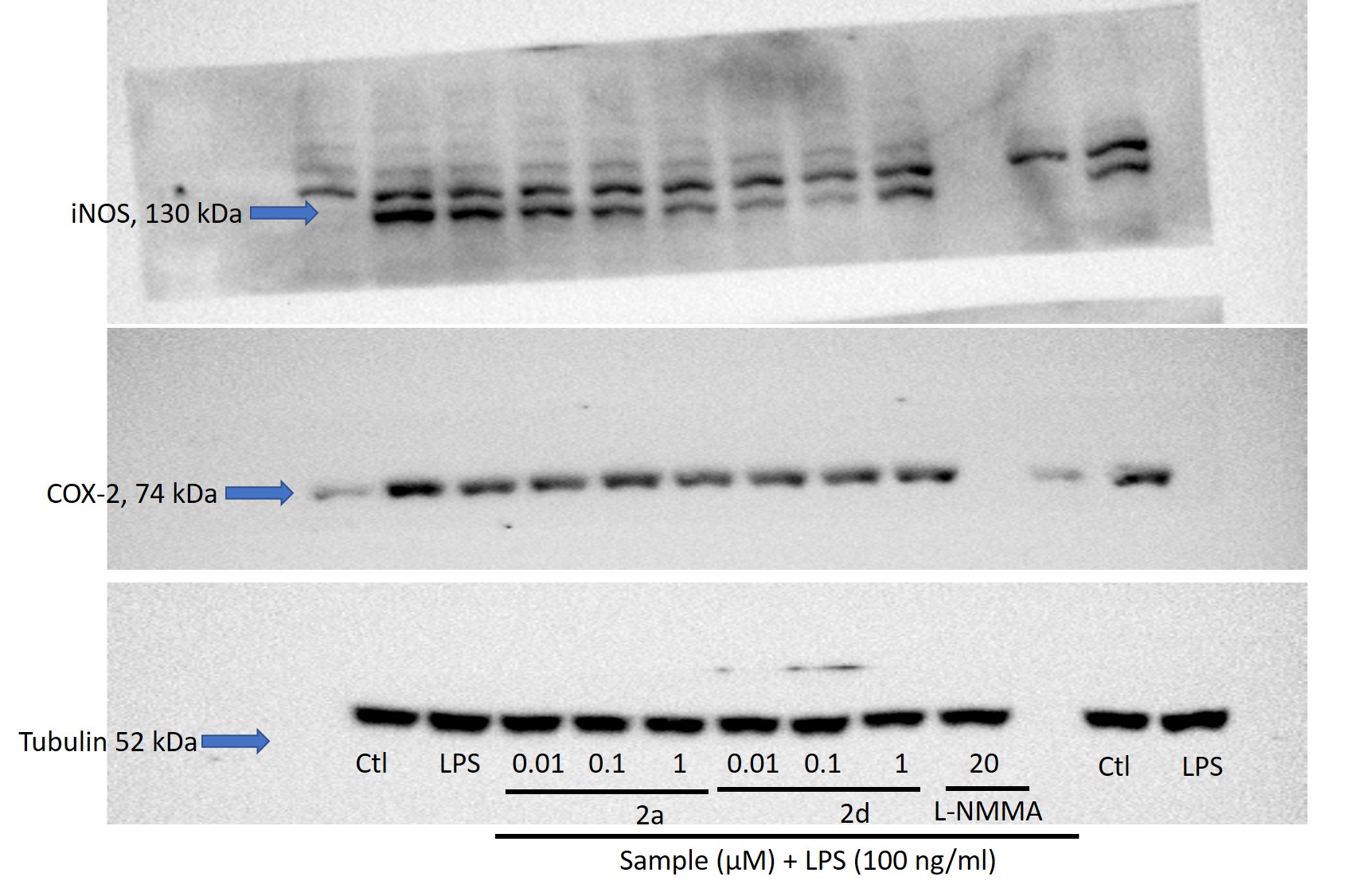


**Supplementary S10:**1H and 13C NMR spectra

^1^H NMR Spectrum of compound 4 in CDCl_3_

^13^C NMR Spectrum of compound 4 in CDCl_3_

^1^H NMR Spectrum of compound 5 in CDCl_3_

^13^C NMR Spectrum of compound 5 in CDCl_3_

^1^H NMR Spectrum of compound 6 in CDCl_3_

^13^C NMR Spectrum of compound 6 in CDCl_3_

^1^H NMR Spectrum of compound 7 in CDCl_3_

^^

^13^C NMR Spectrum of compound 7 in CDCl_3_

^1^H NMR Spectrum of compound 2a in CDCl_3_

^13^C NMR Spectrum of compound 2a in CDCl_3_

^1^H NMR Spectrum of compound 2b in CDCl_3_

^13^C NMR Spectrum of compound 2b in CDCl_3_

^1^H NMR Spectrum of compound 2c in CDCl_3_

^13^C NMR Spectrum of compound 2c in CDCl_3_

^1^H NMR Spectrum of compound 2d in CDCl_3_

^13^C NMR Spectrum of compound 2d in CDCl_3_

^1^H NMR Spectrum of compound 2e in CDCl_3_

^13^C NMR Spectrum of compound 2e in CDCl_3_

^1^H NMR Spectrum of compound 2f in CDCl_3_

^13^C NMR Spectrum of compound 2f in CDCl_3_

^1^H NMR Spectrum of compound 2g in CDCl_3_

^13^C NMR Spectrum of compound 2g in CDCl_3_

^1^H NMR Spectrum of compound 2h in CDCl_3_

^13^C NMR Spectrum of compound 2h in CDCl_3_

__

^1^H NMR Spectrum of compound 2i in CDCl_3_

__

^13^C NMR Spectrum of compound 2i in CDCl_3_

^1^H NMR Spectrum of compound 2j in CDCl_3_

^13^C NMR Spectrum of compound 2j in CDCl_3_

^1^H NMR Spectrum of compound 2k in CDCl_3_

^13^C NMR Spectrum of compound 2k in CDCl_3_

^1^H NMR Spectrum of compound 2l in CDCl_3_

^13^C NMR Spectrum of compound 2l in CDCl_3_

^1^H NMR Spectrum of compound 2m in CDCl_3_

^13^C NMR Spectrum of compound 2m in CDCl_3_

^1^H NMR Spectrum of compound 2n in CDCl_3_

^13^C NMR Spectrum of compound 2n in CDCl_3_

^1^H NMR Spectrum of compound 2o in CDCl_3_

^13^C NMR Spectrum of compound 2o in CDCl_3_

^1^H NMR Spectrum of compound 2p in CDCl_3_

^13^C NMR Spectrum of compound 2p in CDCl_3_

^1^H NMR Spectrum of compound 2q in CDCl_3_

^13^C NMR Spectrum of compound 2q in CDCl_3_

__

^1^H NMR Spectrum of compound 2r in CDCl_3_

^13^C NMR Spectrum of compound 2r in CDCl_3_
